# Inhibition of the m^6^A reader IGF2BP2 as a strategy against T-cell acute lymphoblastic leukemia

**DOI:** 10.1038/s41375-022-01651-9

**Published:** 2022-08-01

**Authors:** Panpan Feng, Dawei Chen, Xia Wang, Yanxia Li, Zhenyu Li, Boya Li, Yupeng Zhang, Wei Li, Jingru Zhang, Jingjing Ye, Baobing Zhao, Jingxin Li, Chunyan Ji

**Affiliations:** 1 Department of Hematology, Qilu Hospital of Shandong University, Jinan, Shandong 250012 China; 2 Department of Physiology, School of Basic Medical Sciences, Cheeloo College of Medicine, Shandong University, Jinan, Shandong 250012 China; 3 Laboratory of Medical Chemistry, GIGA-Stem Cells, Interdisciplinary Cluster for Applied Genoproteomics, Faculty of Medicine, University of Liège, CHU, Sart-Tilman, Liège, 4000 Belgium; 4 Laboratory of Translational Gastroenterology, Department of Gastroenterology, Qilu Hospital of Shandong University, Jinan, Shandong 250012 China; 5 Department of Pharmacology, School of Pharmaceutical Sciences, Cheeloo College of Medicine, Shandong University, Jinan, Shandong 250012 China; 6 Department of Pharmacy, Shandong Provincial Hospital Affiliated to Shandong First Medical University, Jinan, Shandong 250012 China; 7 Department of molecular plant biology, Norwegian Institute of Bioeconomy Research, Oslo, 1431 Ås Norway; 8 Key Laboratory of Chemical Biology (Ministry of Education), School of Pharmaceutical Sciences, Cheeloo College of Medicine, Shandong University, Jinan, Shandong 250012 China

**Keywords:** Acute lymphocytic leukaemia, Oncogenes, Targeted therapies

## Abstract

T-cell acute lymphoblastic leukemia (T-ALL) is an aggressive malignant leukemia with extremely limited treatment for relapsed patients. N6‐methyladenosine (m^6^A) reader insulin-like growth factor 2 mRNA-binding protein 2 (IGF2BP2) participates in the initiation and growth of cancers by communicating with various targets. Here, we found IGF2BP2 was highly expressed in T-ALL. Gain and loss of IGF2BP2 demonstrated IGF2BP2 was essential for T-ALL cell proliferation in vitro and loss of IGF2BP2 prolonged animal survival in a human T-ALL xenograft model. Mechanistically, IGF2BP2 directly bound to T-ALL oncogene NOTCH1 via an m^6^A dependent manner. Furthermore, we identified a small-molecule IGF2BP2 inhibitor JX5 and treatment of T-ALL with JX5 showed similar functions as knockdown of IGF2BP2. These findings not only shed light on the role of IGF2BP2 in T-ALL, but also provide an alternative γ‑Secretase inhibitors (GSI) therapy to treat T-ALL.

## Introduction

T-cell acute lymphoblastic leukemia (T-ALL) is an aggressive hematological neoplasm that frequently occurs in children and adolescents worldwide. T-ALL originates from genomically altered and/or epigenetically changed transformation of immature T cells [1]. Although the current cure rates increased to 80% in children and 60% in adults [2], patients with primary resistant T-ALL frequently fail to obtain a complete hematological remission or relapse after the initial response. This clinical challenge has led researchers to decipher the molecular mechanism of T-ALL transformation and progression and develop alternative drugs to treat this malignancy.

A genome-wide association study of T-ALL cases has identified considerable gene mutations; among them the key oncogenic regulator is NOTCH1, which is found in more than 60% of T-ALL [3]. NOTCH1 activation is triggered by interaction with its ligands, which are members of the Delta and Jagged families and are expressed on neighboring cell surfaces. The activation of the NOTCH1 pathway is essential for early T cell fate determination in the haematopoietic system [4]. Aberrant constitutively active NOTCH1 upregulates anabolic pathways and oncogene MYC expression, which promote T cell leukemogenesis [5]. Nevertheless, inhibiting NOTCH1 signaling using γ‑Secretase inhibitors (GSI) has demonstrated poor efficacy in the clinical treatment of T-ALL [6]. Thus, the identification of other NOTCH1 related genes has become a major priority for the development of anti-NOTCH1 therapies in the clinic.

Posttranscriptional modifications of mRNAs, including N6-methyladenosine (m^6^A), which is the most common internal RNA modification, is well known for regulating gene expression by altering mRNA splicing, stability, translocation, and translation [7]. The methyltransferase complex, which includes the methyltransferase-like 3 and 14 proteins (METTL3 and METTL14) and their regulator Wilms tumor 1-associated protein (WTAP), deposits m^6^A modification, which is then removed by the following “erasers” demethylases: fat mass and obesity-associated protein (FTO) and ketoglutarate- dependent dioxygenase AlkB homolog 5 (ALKBH5) [8]. In addition, the m^6^A modification relies on “readers” to exert its biological function. IGF2BP2, as an m^6^A “reader”, binds RNA through six RNA-binding domains, two of which contain RNA recognition motifs (RRM1 and RRM2) and four KH domains (KH1-KH4) [9]. The KH domains are responsible for the recognition and binding of some specific mRNAs, including ACTIN, MYC and IGF2 [10, 11]. Furthermore, Huilin Huang et al. showed that the KH domains, especially the KH3-4 di-domain, are critical for the binding of IGF2BP2 to m^6^A-modified RNAs [12]. While great effort has been expanded to explore the function of m^6^A modifications in acute myeloid leukemia [13], knowledge regarding the role of IGF2BP2 in T-ALL is limited.

Here we found that IGF2BP2 promoted human T-ALL growth, reduced cytarabine (Ara-C)-, vincristine (VCR)-, venetoclax- or dexamethasone (Dex)-induced apoptosis in vitro and is essential for leukemogenesis in vivo. Further analysis showed that IGF2BP2 could directly bind NOTCH1 and stabilize NOTCH1 mRNA in an m^6^A-dependent manner. In addition, we identified the small molecule JX5 as an IGF2BP2 inhibitor that prevented T-ALL leukemogenesis.

## Materials and methods

### iRIP-seq library preparation and sequencing

IGF2BP2-bound RNA was isolated from immunoprecipitated anti-IGF2BP2 antibody using TRIzol (Invitrogen). Complementary DNA (cDNA) libraries were prepared with a KAPA RNA Hyper Prep Kit (KAPA, KK8541) according to the manufacturer’s procedure and high-throughput sequencing of the cDNA libraries was performed on an Illumina Xten platform for 150 bp paired-end sequencing. After the reads were aligned onto the genome with TopHat 2 [14], only uniquely mapped reads were used for the following analysis. The “ABLIRC” strategy was used to identify the binding regions of IGF2BP2 on the genome [15]. Reads with at least 1 bp overlap were clustered as peaks. For each gene, a computational simulation was used to randomly generate reads with the same number and lengths as the reads in peaks. The output reads were further mapped to the same genes to generate a random max peak height from overlapping reads. The whole process was repeated 500 times. All observed peaks with heights higher than those of the random max peaks (p value < 0.05) were selected. The IP and input samples were analyzed by the simulation independently, and the IP peaks that overlapped with the input peaks were removed. The target genes of IP were finally determined by the peaks and the binding motifs of the IP protein were called by HOMER software [16].

### MeRIP-qPCR

RNA immunoprecipitation was performed as previously described [17]. An m^6^A antibody was used to pull down m^6^A-modified individual RNA. Briefly, the total RNA was extracted by an EASYspin Plus kit (Yishan, China) and dissolved in 40 µL of RNase-free water. Then, the cells were divided into two groups, one group for input and the other group was increased to 1 mL by the addition of buffer containing an RNase inhibitor, ribonucleoside vanadyl complexes, an m^6^A-specific antibody (Sigma–Aldrich) or rabbit IgG (Sigma Aldrich) and incubated for 2 h at 4 °C. Next, the prewashed beads A were added and incubated for 2 h. Elution buffer containing an anti-m^6^A antibody (Synaptic Systems) was added to the mixture and incubated for 1 h with continuous shaking at 4 °C. The methylated mRNA was precipitated with 5 mg of glycogen and one-tenth volumes of 3 M sodium acetate in a 2.5 volume of 100% ethanol at −80 °C overnight. The m^6^A-bound RNA was calculated by qPCR, and the corresponding m^6^A enrichment was calculated by normalizing to the input.

### Molecular docking

A molecular docking study was performed to investigate the binding mode between the compounds and human IGF2BP2 as previously described [18]. The three-dimensional (3D) structure of IGF2BP2 (PDB ID: 6ROL) was downloaded from the RCSB Protein Data Bank (www.rcsb.org). The 2D structures of the compounds were drawn by ChemBioDraw Ultra 14.0 and converted to 3D structures by ChemBio3D Ultra 14.0 software. The AutoDockTools 1.5.6 package [19, 20] was employed to generate the docking input files. The ligands were prepared for docking by merging nonpolar hydrogen atoms and defining rotatable bonds. The search grid of the IGF2BP2 site was identified as center_x: 31.471, center_y: −4.855, and center_z: 11.12 with the following dimensions size_x: 37.5, size_y: 41.25, and size_z: 37.5. To increase the docking accuracy, the value of exhaustiveness was set to 16. For Vina docking, the default parameters were used if they are not mentioned. A SPECS library (http://www.specs.net, approximately 300,000 compounds) was selected for screening using the above IGF2BP2 model. The best-scoring pose as judged by the Vina docking score was chosen and visually analyzed using PyMoL 1.7.6 software (www.pymol.org).

## Results

### IGF2BP2 is highly expressed in T-ALL

**Fig. 1:**
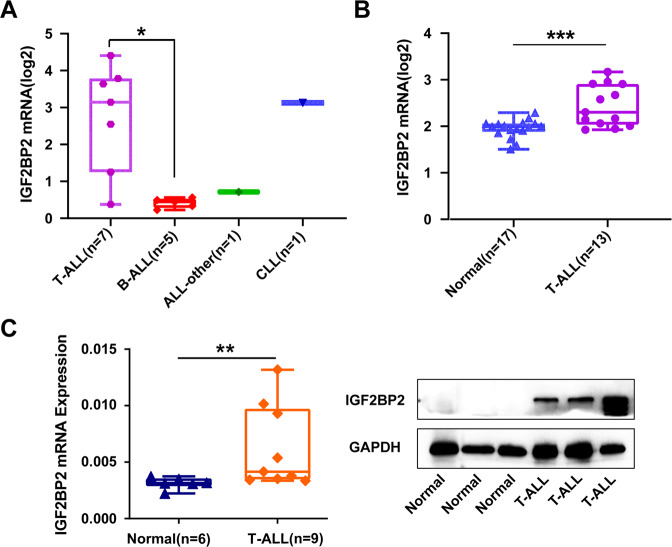
IGF2BP2 is highly expressed in T-ALL. **A** IGF2BP2 expression levels were analyzed among 14 human lymphoblastic leukemia cell lines in CCLE database (https://portals.broadinstitute.org/ccle). **B** IGF2BP2 expression levels in normal T cells (*n* = 17) and primary T-ALL patients cells (*n* = 13) from GEO database, GSE48558. **C** IGF2BP2 expression levels in T-ALL. IGF2BP2 mRNA expression levels (left) were analyzed in normal human bone marrow cells (*n* = 6) and primary T-ALL patients cells (*n* = 9) by RT-qPCR assays. IGF2BP2 protein expression levels (right) were assessed in normal human bone marrow cells (*n* = 3) and primary T-ALL patients cells (*n* = 3) by western blot. Data are mean ± SD values. *P < 0.05; **P < 0.01; ***P < 0.001.

To investigate the potential clinical value of IGF2BP2 in lymphoblastic leukemia, we first explored IGF2BP2 expression by analysing human lymphoblastic leukemia cell lines in the Cancer Cell Line Encyclopedia database (CCLE, https://portals.broadinstitute.org/ccle) [21]. Compared with other lymphoblastic leukemia cell lines, such as B-cell acute lymphoblastic leukemia (B-ALL), and chronic lymphoblastic leukemia (CLL), IGF2BP2 was the most highly expressed in T-cell acute lymphoblastic leukemia (T-ALL) among 14 human lymphoblastic leukemia cell lines (Fig. 1A). To determine IGF2BP2 expression in T-ALL patients, the assessment of human leukemia databases (GSE48558) showed a significant increase in IGF2BP2 and IGF2BP3 expression in T-ALL cells compared with that in normal T cells, whereas YTHDC1, YTHDC2 and METTL16 were decreased; IGF2BP1 or other m^6^A modification enzymes were no significantly different (Fig. 1B, Supplementary Fig. 1A and Supplementary Fig. 2). In addition, we analyzed primary bone marrow mononuclear cells from T-ALL patients and controls. IGF2BP2 mRNA expression (Fig. 1C, left) significantly increased in T-ALL (*n* = 9) compared to that in the control (*n* = 6), but not in IGF2BP1 and IGF2BP3 (Supplementary Fig. 1B). Consistently, the T-ALL (*n* = 3) patients had a higher IGF2BP2 protein expression (Fig. 1C, right & Supplementary Fig. 1C) than the control patients (*n* = 3). Moreover, we evaluated m^6^A modification enzymes expression in different molecular subtypes of T-ALL from PeCan database and NCI TARGET database (phs000464). IGF2BP2 displayed higher expression levels in HOXA, KMT2A and NOTCH1 mutation subtypes compared with other T-ALL subtypes (Supplementary Fig. 3). Taken together, we identified a global upregulation of IGF2BP2 in T-ALL.

### IGF2BP2 promotes cell survival and impairs chemotherapy sensitivity in T-ALL

**Fig. 2:**
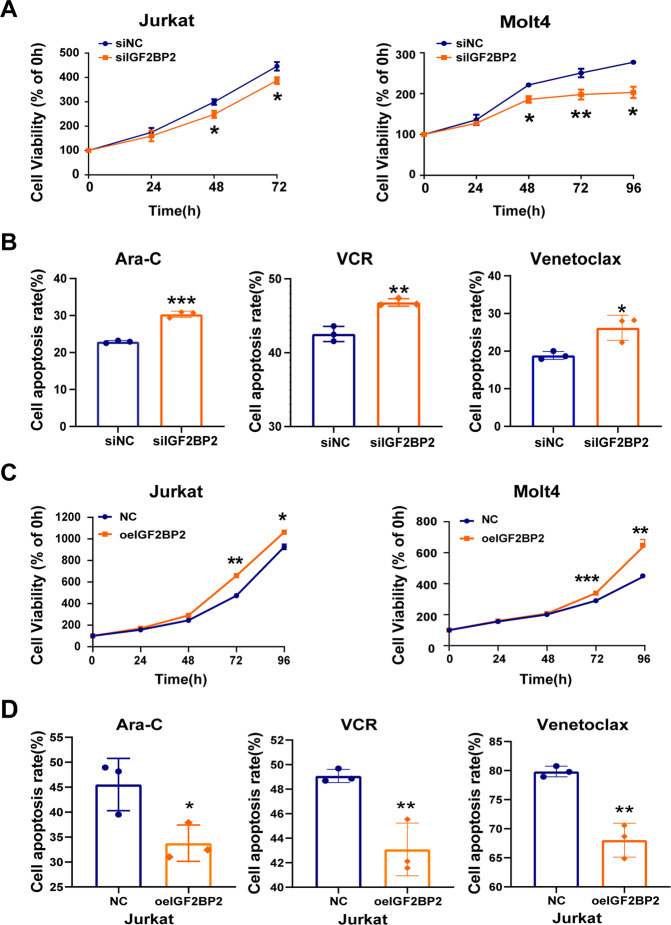
IGF2BP2 promotes cell survival and impairs chemotherapy sensitivity in T-ALL. **A** Proliferation of Jurkat cells (siIGF2BP2 or siNC) and Molt4 cells (siIGF2BP2 or siNC) was assessed by CCK8 assays, and proliferation rates at 0, 12, 24, 48, 72 and 96 h were normalized to the absorbance at 0 h. **B** Apoptosis analysis of Jurkat cells (siIGF2BP2 or siNC) after 24 h treatment with Ara-C, VCR and venetoclax were measured by flow cytometry. Percentages were representative of cell apoptosis from three replicate experiments. **C** Proliferation of Jurkat cells (oeIGF2BP2 or NC) and Molt4 cells (oeIGF2BP2 or NC) were assessed by CCK8 assays, and proliferation rates at 0, 12, 24, 48, 72 and 96 h were normalized to the absorbance at 0 h. **D** Apoptosis analysis of Jurkat cells (oeIGF2BP2 or NC) after 24 h treatment with Ara-C, VCR and venetoclax were measured by flow cytometry. Percentages were representative of cell apoptosis from three replicate experiments. Data are mean ± SD values. *P < 0.05; **P < 0.01; ***P < 0.001.

To further explore the role of IGF2BP2 in T-ALL, we downregulated IGF2BP2 expression in Jurkat and Molt4 cells by siRNA. The suppression of IGF2BP2 expression was verified by RT-qPCR and western blots analysis (Supplementary Fig. 4A). We found that the IGF2BP2 downregulation markedly inhibited cell growth by CCK8 assays (Fig. 2A) and cell cycle arrest by a propidium iodide (PI) kit (Supplementary Fig. 4B). And cell apoptosis was increased after the IGF2BP2 knockdown (Supplementary Fig. 4C). Additionally, the IGF2BP2 downregulation increased the Ara-C-, VCR- and venetoclax-induced cell apoptosis (Fig. 2B). In contrast, the overexpression of IGF2BP2 by specific lentivirus (Supplementary Fig. 4D) promoted the proliferation of Jurkat and Molt4 cells (Fig. 2C). The upregulation of IGF2BP2 raised the IC50 of chemotherapeutics in Jurkat and Molt4 cell (Supplementary Fig. 4E). Similarly, the IGF2BP2 overexpression decreased Ara-C-, VCR-,venetoclax- and Dex-induced cell apoptosis (Fig. 2D & Supplementary Fig. 4F), confirming that IGF2BP2 could promote T-ALL resistant to chemotherapeutics, including Ara-C, Dex, VCR and venetoclax. Our data indicate that IGF2BP2 was sufficient to confer cell survival and chemo-resistance in T-ALL.

### Transcriptome-wide iRIP-sequence assays identify potential targets of IGF2BP2 in T-ALL

**Fig. 3:**
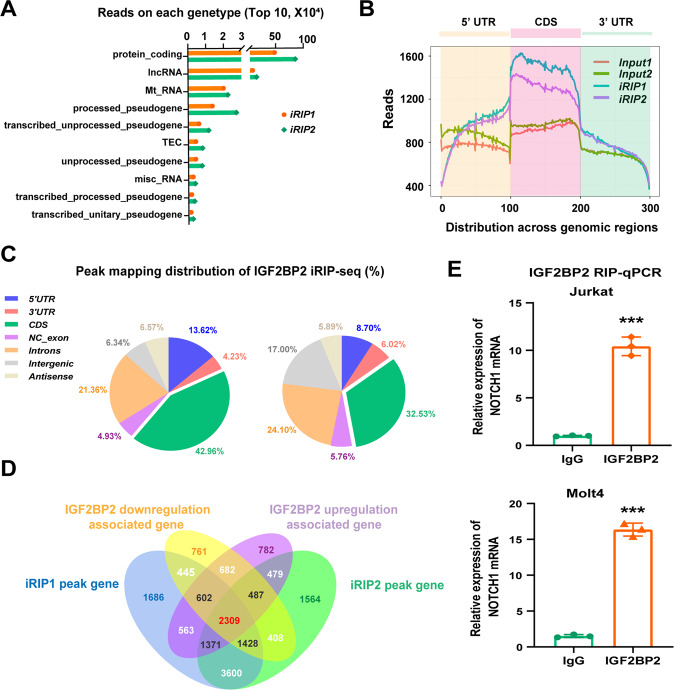
Transcriptome-wide iRIP-sequence assays identifies potential targets of IGF2BP2 in T-ALL. **A** The Top 10 RNA reads within different gene types bound by IGF2BP2 from two replicate IGF2BP2 binding iRIP-sequence data. Protein_coding, contains an open reading frame (ORF); for detailed explanations of other gene types, refer to following website, https://www.gencodegenes.org/pages/biotypes.html. **B** Metagene profiles of enrichment of IGF2BP2-binding sites and their Input control analyzed by two replicate iRIP-sequence. CDS, coding sequence. **C** The distribution of IGF2BP2-binding peaks within different gene regions from two replicate IGF2BP2 binding iRIP-sequence data. **D** Venn diagram was plotted to show the intersected genes from IGF2BP2 binding iRIP-sequence, RNA-sequence of IGF2BP2 downregulation associated gene and IGF2BP2 upregulation associated gene. 2309 common genes were screened out. **E** The enrichment of NOTCH1 was analyzed by IGF2BP2 RIP-qPCR in Jurkat and Molt4 cells. Data are mean ± SD values. *P < 0.05; **P < 0.01; ***P < 0.001.

Next, we tried to explore the potential targets involved in IGF2BP2-mediated T-ALL cell survival. Considering the properties of IGF2BP2 as an mRNA binding protein [9], we subsequently explored the transcriptome-wide binding profile of IGF2BP2 in Jurkat cells by employing an improved RNA immunoprecipitation-coupled high-throughput sequencing (iRIP-seq) approach, a recently developed UV-crosslinking immunoprecipitation method [17]. The Gene Ontology (GO) analysis of iRIP-seq showed that IGF2BP2 was involved in RNA binding in T-ALL cells (Supplementary Fig. 5A). The data of the top ten gene types in two replicate iRIP-seq experiments derived from read numbers bound by the IGF2BP2 protein revealed that the majority of IGF2BP2-binding reads were located in protein-coding RNA (i.e., mRNA) (Fig. 3A). Moreover, most IGF2BP-binding reads (Fig. 3B) and peaks (Fig. 3C) were located in protein-coding transcripts and were highly enriched in the coding sequence (CDS) of mRNA. Then we performed RNA sequence of in IGF2BP2 up- or down-regulated Jurkat cells. Combining the IGF2BP2 downregulation associated protein-coding RNA and IGF2BP2 upregulation associated protein-coding RNA, we found overlap with our two replicate iRIP-seq data, and finally identified 2309 common genes (Fig. 3D and supplementary file 1). Among these overlapping genes, we noticed that plenty of genes (NOTCH1, NKX2-1, BCL11B, RUNX1, FBXWT, STAT5B, DNM2, AKT1/2, MTOR, ABL1, DNMT3A, and RPL11) were associated with T-ALL pathogenesis(Supplementary Fig. 5B) [22]. Among these genes, NOTCH1 was a striking oncogenic driver of T-ALL [23]. Intuitive histogram showed the link between IGF2BP2 and NOTCH1 mRNA from two independent iRIP sequence (Supplementary Fig. 6A).

Next, we determined whether NOTCH1 could be a target of IGF2BP2 in T-ALL. RNA immunoprecipitation-coupled qPCR (RIP-qPCR) with an IGF2BP2 antibody was used to measure the NOTCH1 mRNA levels bound by IGF2BP2 in Jurkat and Molt4 cells (Fig. 3E), suggesting that IGF2BP2 could directly bind NOTCH1 mRNA. Additionally, we performed a western blot analysis of IGF2BP2 suppression or knockdown cells and found that the NOTCH1 protein levels were downregulated compared to those in the control cells (Supplementary Fig. 6B). In contrast, IGF2BP2 overexpression upregulated the NOTCH1 protein levels (Supplementary Fig. 6C). Then, we performed a rescue experiment of IGF2BP2 inhibition in NOTCH1 overexpressed cells. As expected, the inhibition of IGF2BP2 resulted in re-downregulation of NOTCH1 (Supplementary Fig. 6D) and alleviated the pro-proliferative function of NOTCH1 overexpression to a certain degree (Supplementary Fig. 6E). Taken together, these findings strongly indicate that IGF2BP2 directly bound NOTCH1 mRNA and promoted NOTCH1 expression to regulate the survival of T-ALL cells.

### IGF2BP2 regulates NOTCH1 mRNA stability through m^6^A modification

**Fig. 4:**
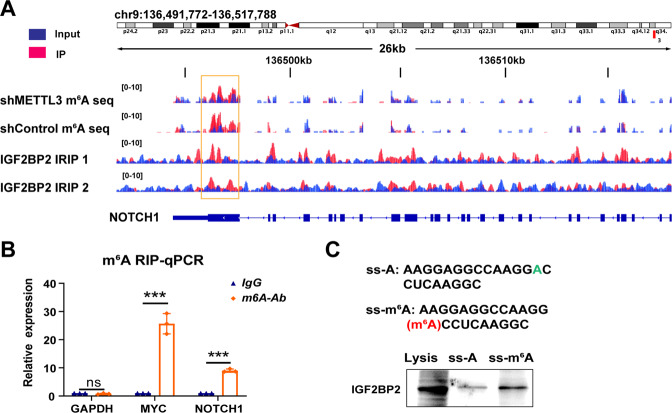
IGF2BP2 enhances NOTCH1 mRNA stability via an m^6^A-dependent manner. **A** Distribution of m^6^A peaks and IGF2BP2-iRIP peaks across NOTCH1 mRNA transcript. The overlap region is highlighted in yellow. m^6^A-seq was repeated once, whereas RIP-sequence was performed twice. **B** M^6^A enrichment of NOTCH1 mRNA in Jurkat cells by m^6^A-RIP-qPCR. Results are presented relative to those obtained with immunoglobulin G (IgG). Glyceraldehyde 3-phosphate dehydrogenase (GAPDH), m^6^A negative control; MYC peak, m^6^A positive control. **C** IGF2BP2 expression was measured by western blot in Jurkat cells after RNA pull down assay using single-stranded NOTCH1 RNA with methylated or unmethylated adenosine. Data are mean ± SD values. *P < 0.05; **P < 0.01; ***P < 0.001.

To further explore the underlying mechanism of IGF2BP2-mediated NOTCH1 expression, we assessed whether IGF2BP2 interacts with NOTCH1 as an N6-methyadeninosine (m^6^A) reader. We performed m^6^A sequencing in control and METTL3-knockdown cells. As shown in Fig. 4A, the m^6^A modifications were accumulated across the NOTCH1 transcript, and the m^6^A peaks coincided well with the IGF2BP2-binding sites identified by iRIP-sequence (yellow box). Notably, the m^6^A modifications decreased remarkably upon METTL3 knockdown (Fig. 4A). Moreover, endogenous IGF2BP2 strongly bound the ‘GGAU/G/A/C’ consensus sequence containing the ‘GGAC’ m^6^A modification core motif (Supplementary Fig. 7A). Then, m^6^A RNA-real-time qPCR with an m^6^A antibody, RIP-qPCR with an IGF2BP2 antibody and gene-specific m^6^A pull down assay were performed. We measured the m^6^A levels of NOTCH1 by m^6^A RIP-qPCR in Jurkat cells showing that m^6^A modified the mRNA of NOTCH1 (Fig. 4B). Similarly, knockdown of METTL3 also reduced the NOTCH1 protein level (Supplementary Fig. 7B). Notably, our data showed that the binding activity between IGF2BP2 and the NOTCH1 mRNA fragment was significantly reduced after METTL3 downregulation by lentivirus (Supplementary Fig. 7C). IGF2BP2 can enhance mRNA stability and translation which are recognized by RNA N6-methyladenosine [24]. The RNA decay assessment revealed that NOCTH1 mRNA decayed faster in the IGF2BP2-downregulated cells than in the control cells (Supplementary Fig. 7D), suggesting that IGF2BP2 could increase NOCTH1 mRNA stability. Through an analysis of the overlap in the binding peaks of m^6^A-seq and iRIP-seq data, we designed methylated single-stranded RNA bait (ss-m^6^A) or unmethylated control RNA (ss-A) for RNA pull-down. Consistent with previous results, the RNA pull-down assay confirmed that the IGF2BP2 protein preferentially bound the methylated bait rather than the unmethylated control (ss-A) of NOTCH1 (Fig. 4C). Taken together, these findings collectively supported that IGF2BP2 served as an m^6^A reader to increase NOTCH1 stability in the processing of methylation.

### Knockdown of IGF2BP2 significantly decreases the leukemia burden in human T-ALL xenografts

**Fig. 5:**
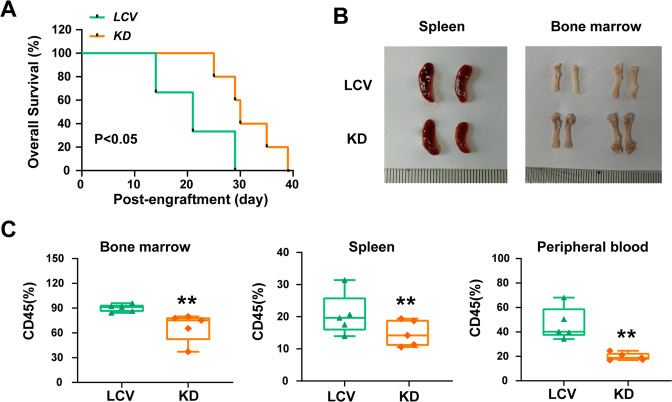
IGF2BP2 knockdown significantly decreases leukemia burden in the human T-ALL xenograft. **A** Kaplan–Meier survival curves of Jurkat xenografts in each group as indicated (*n* = 7 for each group). Survival of KD or LCV2 mice (orange and green lines) are compared with p value provided as shown (log-rank test). **B** Representative spleen and bone images of mice carrying Jurkat cells with or without IGF2BP2 expression. **C** Human CD45^+^ cells from bone marrow, spleen and peripheral blood were analyzed by flow cytometry. Data from five individual mice were plotted and shown on the right. Data are mean ± SD values. *P < 0.05; **P < 0.01; ***P < 0.001.

To assess the role of IGF2BP2 in leukemogenesis, we established IGF2BP2 knockdown and control Jurkat cell lines, using LCV-2m IGF2BP2-KD virus or LCV-2m control virus packaged by the Pkat/VSVG system and transplanted these cells into immunocompromised M-NSG mice (Supplementary Fig. 8A). The IGF2BP2 suppression in Jurkat cells was verified by a western blot analysis (Supplementary Fig. 8B). We regularly monitored the mice leukemia progression, and leukemia burden was evaluated by assessing the degree of organomegaly and T-ALL cell infiltration. Two weeks later emaciation and tetraplegia, which are indications of disease onset and progression were observed in the two groups. In contrast, the IGF2BP2 suppression significantly improved the animal survival rates and prolonged the lifespan of the mice, showing that IGF2BP2 was associated with T-ALL leukemogenesis (Fig. 5A). Consistently, the mice bearing IGF2BP2-deficient cells manifested ameliorated splenomegaly, more reddish bones (Fig. 5B), and suppressed leukemia cell dissemination in the bone marrow and spleen (Supplementary Fig. 8C). The flow cytometry staining confirmed that the IGF2BP2 deficiency significantly decreased the human CD45^+^ leukemia burden in the bone marrow, spleen and peripheral blood (Fig. 5C & Supplementary Fig. 8D). Overall, these findings demonstrated that IGF2BP2 played a vital role in T-ALL initiation and progression.

### Small-molecule inhibition of IGF2BP2 as a strategy against T-ALL

To investigate the therapeutic potential of IGF2BP2 inhibition in T-ALL, we attempted to develop a small molecular inhibitor of IGF2BP2. Given that KH domains, especially the KH3-4 di-domain, are critical for the binding of IGF2BP2 to m^6^A-modified RNAs [12], we performed virtual high throughput-screening using a molecular docking model with the three-dimensional structure of the IGF2BP2 (PDB ID: 6ROL). We first screened approximately 300,000 small molecule compounds, and identified 22 hit compounds that potentially bind IGF2BP2 KH3-4 domains (Supplementary file 2). These hit compounds were subsequently screened using an internal fluorescence quenching assay, which revealed real biophysical association between 4 hit compounds and IGF2BP2 (Supplementary Fig. 9A).

**Fig. 6:**
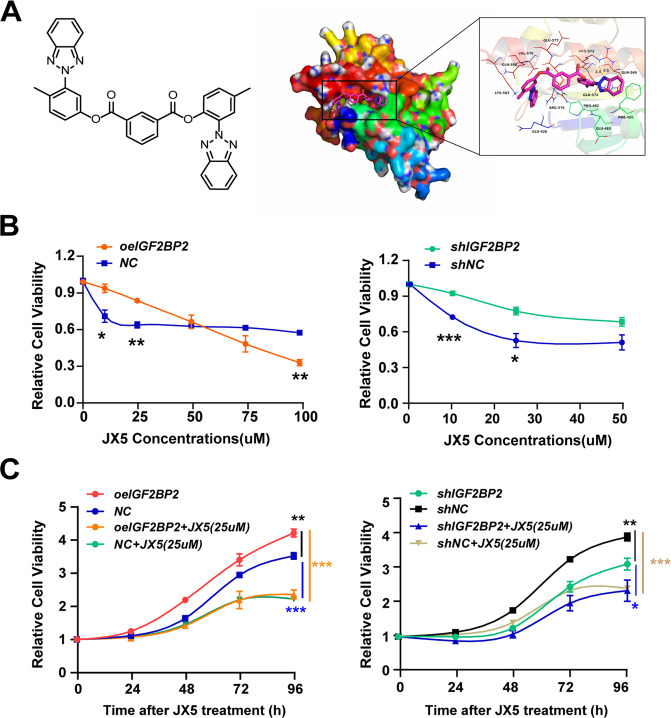
Small-molecule inhibitor of IGF2BP2 as a strategy against T-ALL. **A** Small-molecule inhibitor JX5. Chemical structure JX5 (left side). 3D (left) and surface (right) view models show the binding pocket and binding of IGF2BP2 and JX5. Molecular docking of JX5 with IGF2BP2 (PDB: 6ROL) were performed (right side). **B** Cell growth inhibition of Jurkat cells (oeIGF2BP2 or NC, shIGF2BP2 or shNC) were assessed by CCK8 assays after 48 h treatment with serial dilutions of JX5. **C** Proliferation of Jurkat cells (oeIGF2BP2 or NC, shIGF2BP2 or shNC) treated with 25uM JX5 were assessed by CCK8 assays, and proliferation rates at 0, 12, 24, 48, 72 and 96 h were normalized to the absorbance at 0 h. Data are mean ± SD values. *P < 0.05; **P < 0.01; ***P < 0.001.

We then evaluated the activity of these 4 hits in Jurkat cells (shcontrol and shIGF2BP2) respectively and found that JX5 had specific inhibitory effects on the proliferation of Jurkat shcontrol cells (Fig. 6A and Supplementary Fig. 9D). JX5 adopted a compact conformation to bind in the site of IGF2BP2 (Fig. 6A). The binding site between JX5 and full-length IGF2BP2 was directly confirmed with a K_d_ value of 93.2 ± 3.9 μM by the internal fluorescence quenching measurement assay (Supplementary Fig. 9B, C). Compared to the mild inhibition of Jurkat cells proliferation and survival due to the naive IGF2BP2 level, JX5 showed dramatic cytotoxicity against Jurkat cells with IGF2BP2 overexpression, whereas it had minor effects on Jurkat cells transduced with IGF2BP2 shRNA (Fig. 6B, C). Consistent with the effect of IGF2BP2 knockdown, JX5 treatment also led to cell apoptosis in Jurkat cells (Supplementary Fig. 9E). Intriguingly, JX5 had no effect on the IGF2BP2 mRNA levels but significantly down regulated the NOTCH1 expression (Supplementary Fig. 9F, G). The protein level of NOTCH1 was also greatly reduced in Jurkat cells in the present of JX5, whereas IGF2BP2 and METTL3 were no significant difference (Supplementary Fig. 9H). Additionally, the m^6^A levels of NOTCH1 was measured by m^6^A RIP-qPCR in JX5 treated Jurkat cells showing that the NOTCH1 mRNA was decreased (Supplementary Fig. 9I). These data demonstrated that JX5 was a novel inhibitor of IGF2BP2, which could suppress the T-ALL cells proliferation via downregulation of NOTCH1 expression.

Following the positive evidence for pharmacological inhibition of IGF2BP2 in vitro, we performed in vivo studies using T-ALL xenografts. Initially, the six-week-old male M-NSG mice were irradiated at 1 Gy before tail vein injection of 5 × 10^6^ Jurkat cells. One week later, daily treatment with vehicle or JX5 (1 mg/kg or 2 mg/kg) by intraperitoneal injection (Supplementary Fig. 10A). Leukemia burden was evaluated by assessing the degree of organomegaly and T-ALL cell infiltration. Similarly, we monitored the mice body weight, pellets, and the structures of small intestine, colon and stomach to evaluate the compound’s gastrointestinal toxicity. Notably, two mice from the vehicle group died on day 16 and day18, respectively, and then all remaining were euthanized. The anti-leukemic effect was confirmed by the reduction of human CD45^+^ cells in the bone marrow and spleen following treatment (Supplementary Fig. 10C, D). Consistently, the mice treated with JX5 manifested ameliorated splenomegaly (Supplementary Fig. 10B). Additionally, JX5 has no overt toxicity or effect on mouse body weight and gastrointestinal system (Supplementary Fig. 10E–H). In summary, the pharmacological inhibition of JX5 in vivo impaired T-ALL expansion, and had no lasting effect on gastrointestinal system.

## Discussion

IGF2BP2 has been implicated in a wide range of cancer types. In triple-negative breast cancer, high IGF2BP2 expression is associated with cancer cell migration and invasion and promotes the stability of MYC mRNA [25]. In colorectal cancer, IGF2BP2 regulates cancer cells glycolysis by controlling hexokinase and glucose transporter 1 expression [26]. In the present study, we showed that IGF2BP2 was aberrantly upregulated in a subset of human T-ALL and contributed to the survival of T-ALL. This notable role of IGF2BP2 attributes to stabilization of NOTCH1 in an m^6^A dependent way, bringing a new layer to the critical function of m^6^A modification in the pathology of T-cell leukemogenesis.

Despite the emerging understanding of the role of m^6^A modifications in various cancer types [27], the underlying function and mechanism in T-ALL are poorly explored. Here, we prove that the m^6^A modification is required for the binding of IGF2BP2 to its target NOTCH1 to promote T-ALL cells against chemoresistance. However, recent study demonstrated that downregulation of the demethylase ALKBH5 ameliorates T-ALL chemoresistance by stabilizing ubiquitin‐specific protease 1 [28]. This paradoxical phenomenon of m^6^A modification function also occurs in other systems; for example, the siRNA knockdown of METTL3 in mouse bone marrow derived macrophages (BMDMs) reduces proinflammatory cytokine production [29], while the loss of IGF2BP2 result in an enhanced proinflammatory phenotype in mouse BMDMs [17]. In addition, different readers can play opposite roles in the same cancer; IGF2BP1 is an important protumorigenic factor in pancreatic cancer [30], nevertheless, YTHDF2 is poor expressed in liver cancer and inhibits cancer cells proliferation and growth [31]. Hence, more studies are needed to discover these divergent roles of m^6^A modifications in T-ALL.

Mutated NOTCH1 plays a significant role in the onset and development of T-ALL. Many small compounds in the NOTCH1 pathway have been investigated as a targeted therapy for T-ALL [32]. As γ-secretase leads to the release of the intracellular domain of Notch1 (ICN1) and inhibition of γ-secretase is able to induce G0/G1 cell cycle arrest and inhibit T-ALL proliferation, plenty of GSIs were put into clinical trials, but failed because of gastrointestinal toxicity and limited anti-leukemia effects [33]. Besides targeting γ-secretase, drugs, that target other NOTCH1 signaling genes have shown efficiency against T-ALL in animal experiments [34, 35]. Due to these potential effects of targeted therapy on the NOTCH1 signaling pathway in T-ALL but none of agents currently applied in clinical practice, we present the characterization of JX5, which could inhibit the binding of IGF2BP2 with NOTCH1 to deactivate NOTCH1 signaling in T-ALL. Though low dosage of JX5 (25 μM) showed the potential therapeutic effect of inhibition in T-ALL similar to that of IGF2BP2 interference (Fig. 6C), it only reduced half of T-ALL cells viability after 4 days treatment, which might provide a slight possibility of surviving T-ALL to evolve into an IGF2BP2-resistant phenotype. To prevent the recurrence of T-ALL, it is expected that further investigation of the combination JX5 with other reagents or therapies could essentially benefit T-ALL patients. However, it is noted that IGF2BP2 interference or JX5 treatment induced apoptosis of human T-ALL cell lines (Supplementary Figs. 4C and 9E), while most GSIs inhibit cells proliferation mainly by arresting cell cycle [33]; additionally, JX5 treatment also affected the NOTCH1 independent T-ALL cell lines LOUCY viability slightly (Supplementary Fig. 9J), implying that inhibiting IGF2BP2 have some non-NOTCH1 effects. Indeed, IGF2BP2 can promote the expression of ErbB2 to repress colon cancer cells apoptosis [36]. Considering that IGF2BP2 is involved in m^6^A modification and this RNA modification influences various cellular activities in cancers [37], further investigations are needed to understand the off-target effects and toxicity of JX5.

## Conclusion

Taken together, we propose a model in which high IGF2BP2 expression maintains and increases the growth of transformed T-ALL cells via the stabilization of T-ALL oncogene NOTCH1. Based on our results and public data, we developed the active IGF2BP2 inhibitor JX5, which effectively suppressed the activation of NOTCH1 and the growth of T-ALL. Thus, JX5 might be beneficial for T-ALL patients.

## Supplementary information

Supplemental material

## Data Availability

The data that support the findings of this study are available from the corresponding author upon reasonable request. Gene Expression Omnibus: newly generated. iRIP-Sequence and MeRIP-Sequence raw data were deposited under accession number GSE205503 and GSE207323.
