# Supplementary material for: Inhibition of the m6A reader IGF2BP2 as a strategy against T-cell acute lymphoblastic leukemia
Source: Leukemia. 2022 Aug 1;36(9):2180–8. doi: 10.1038/s41375-022-01651-9 (PMC9417996; doi:10.1038/s41375-022-01651-9)
Supplement: Supplementary file 1 — Supplemental material [file 41375_2022_1651_MOESM1_ESM.docx]

**Materials and Methods**

**Patient samples:** Bone marrow (BM) samples from 9 T-ALL patients and 6 control donors were obtained at the Qilu Hospital of Shandong University, China. Control samples were obtained from donors without any malignant bone marrow disorder. Informed consent was obtained in accordance with the Declaration of Helsinki. Bone marrow samples were acquired with the informed consent of the patients. The use of human tissues was approved by the Medical Institutional Ethics Committee of Qilu Hospital, Shandong University (NO. KYLL-2017(KS)-197), China. Bone marrow mononuclear cells were isolated by density gradient centrifugation using Lymphocyte separation medium (Haoyang, China).

**Reagents:** The antibodies against IGF2BP2 (11601-1-AP) and GAPDH (60004-1-Ig) for the western blot analysis were purchased from Proteintech (Wuhan, China). The antibodies NOTCH1 (4147) and METTL3 (86132) for the western blot analysis were purchased from Cell Signaling Technology (USA). The antibodies IGF2BP2 (#A500-012A) for RIP-sequence and RIP-qPCR were purchased from Bethyl Company (USA). The antibodies against CD45 (304012) for flow cytometry were purchased from Biolegend Technology (US). Lentiviral constructs expressing IGF2BP2 were purchased from Genechem (Shanghai, China), and those expressing cleaved NOTCH1 were purchased from Genepharma (Shanghai, China). The compounds Ara-C (HY-13605), Dex ([HY-14648](https://www.medchemexpress.cn/Dexamethasone.html)), venetoclax (HY-15531) were purchased from MedchemExpress (USA). The compounds vincristine (VCR, GN10250) was purchased from GLPbio (USA). The siRNA sequence targeting IGF2BP2 was designed and synthesized by Hippobio Biotechnology Company (Huzhou, China). Primers were purchased from Biosune Biotechnology Company (Shanghai, China).

**Cell lines and culture:** The human leukemic cell lines Jurkat and Molt4 were purchased from the Institute of Hematology and Blood Diseases Hospital, Chinese Academy of Medical Sciences and Peking Union, Medical College, Tianjin, China. The Jurkat and Molt4 cells were grown at 37℃, 5% CO_2_ in RPMI-1640 medium (Gibco, USA) supplemented with 10% foetal bovine serum (FBS, Gibco, USA) and 1% penicillin/streptomycin, respectively. These two cell lines were authenticated by a short tandem repeat analysis (Zsbio company, Shanghai) and were conventionally assessed for mycoplasma contamination by RT**–**qPCR.

**Total RNA isolation and RT–qPCR:** Total RNA was isolated using TRIzol (TaKaRa, Japan) according to the manufacturer’s protocols. The RNA (0.5 μg) was reverse transcribed into cDNA using an RT reagent kit (TaKaRa, Japan). Quantitative real-time polymerase chain reactions were performed by an Applied Biosystem 7900HT System (ABI) with appropriate primers, using SYBR Green PCR Master Mix Vazyme, China). The human housekeeping gene GAPDH was used as the RNA-loading control. The △△Ct values were normalized to GAPDH, and the relative quantification of gene expression was compared to the control group.

**siRNA transfection:** For the transfection of siRNA, 5 × 10^5^ cells were plated per well in a 6-well plate, and transfected with 10 μl INTERFERin (Polyplus) and 50 nmol IGF2BP2 siRNA or control siRNA according to the manufacturer’s instructions. The cells were collected on three consecutive days and further experiments were performed 48 h or 72 h after the first transfection.

**Lentiviral transduction:** Lentiviral constructs expressing IGF2BP2 were purchased from Genechem (Shanghai, China). The IGF2BP2 KD was performed as previously described ^[14]^. We used LCV2-m IGF2BP2-KD, LCV2-m control, Pkat and VSVG packaging plasmids to construct the lentivirus in 293T cells. 1 × 10^7^ 293T cells were plated per well in a 10 cm plates, and then, a total of 20 ug plasmids (LCV2-m IGF2BP2-KD/ LCV2-m control : Pkat : VSVG, 3 : 2 : 10) companied with 40 ul Lipofectamine 2000 (Invitrogen, USA) were carefully added to the plates at 24 hours. The medium containing the target lentivirus were collected at 48 and 72 hours. We introduced the target lentivirus or control lentivirus into Jurkat and Molt4 cells, seeded in 24-well plates at 5×10^4^ cells. Three days later, the infected cells were subjected to puromycin (1 μg/ml) selection for 7 days. Individual puromycin-resistant colonies were manually selected and then expanded in 6-well plates.

**Western blot analysis:** The cells were washed three times with cold PBS and lysed in protein lysis buffer (BestBio, China) with a protease and phosphatase inhibitor mixture (BestBio, China) for 20 min, and then centrifuged at 12000 rpm, 4 ℃. The protein concentration was quantified by a bicinchoninic acid (BCA) protein kit (Beyotime, China). The protein expression levels were determined by staining with primary antibodies and relevant secondary (1:5000, Zsbio, China) antibodies. The primary antibodies (anti-IGF2BP2, anti-NOTCH1 and anti-METTL3) were diluted 1:1000 in 5% fat-free milk. Anti-β-actin (1:1000, Zsbio, China) was used as a loading control. The protein bands were visualized using a FluorChem E Chemiluminescent Western Blot Imaging System (Cell Biosciences).

**Cell proliferation assay and IC50:** Cell proliferation was analyzed using CCK8 assays (BestBio, China) according to the manufacturer’s instructions. Briefly, Jurkat or Molt4 cells were seeded in 96-well plates at a density of 3-5×10^3^ cells per well. Then, 10 µl CCK-8 were added to the wells at 0, 24, 48, 72, and 96 hours. The absorbance at 450 nm was measured after incubation at 37 °C for 2-4 h. To measure the half maximal inhibitory concentration (IC50), the cells were exposed to serial dilutions of cytarabine (Ara-C) or dexamethasone (Dex) for 48 h. The cell viability was determined by CCK-8 assays, and the IC50 values were calculated.

**Cell cycle assay:** For the cell cycle assay, 1×10^6^ cells were plated per well in a 6-well plate. After interference with IGF2BP2 siRNA for a specified time, 400 μL PI were added to the cell suspension. The samples were filtered through 200-mesh filters and then analyzed using a flow cytometer. The DNA content of the cells was analyzed to determine the percentage of cells in the G0/G1, S and G2/M phases.

**Cell apoptosis assay:** For the cell cycle assay, 1×10^6^ cells were plated per well in a 6-well plate, treated with Ara-C, Dex, vincristine (VCR), venetoclax and JX5 for 24 h or 48 h, and stained with Annexin V/7-AAD (BestBio, China) for 15 min at room temperature in the dark according to the manufacturer’s instructions. Apoptosis was analyzed by flow cytometry (Beckman Coulter).

**RNA extraction and sequencing:** The total RNA was extracted with TRIzol (Ambion). The RNA was further purified with two phenol–chloroform treatments and then treated with RQ1 DNase (Promega, Madison, WI, USA) to remove DNA. The quality and quantity of the purified RNA were redetermined by measuring the absorbance at 260 nm/280 nm (A260/A280) using SmartSpec Plus (BioRad, USA). The integrity of the RNA was further verified by 1.5% agarose gel electrophoresis. For each sample, 1 μg of the total RNA was used for the RNA-seq library preparation by a VAHTS Stranded mRNA-sequence Library Prep Kit (Vazyme, China). Polyadenylated mRNAs were purified, fragmented and then converted into double-stranded cDNA. After end repair and A tailing, the DNA was ligated to VAHTS RNA Adapters (Vazyme, China). Purified ligation products corresponding to 200-500 bps were digested with heat-labile UDG, and the single strand cDNA was amplified, purified, quantified and stored at -80 °C before sequencing. For the high-throughput sequencing, the libraries were prepared following the manufacturer's instructions and applied to an Illumina HiSeq X Ten system for 150 nt paired-end sequencing.

**Coimmunoprecipitation:** Jurkat cells were first lysed in ice-cold lysis buffer (1×PBS, 0.5% sodium deoxycholate, 0.1% SDS, and 0.5% NP40) with an RNase inhibitor (Takara, USA) and a protease inhibitor (Solarbio, China) on ice for 5 min. The mixture was then vigorously vibrated and centrifuged at 13,000 x g at 4 °C for 20 min to remove cell debris. The supernatant was incubated with DynaBeads protein A/G (Thermo, USA) conjugated with an anti-IGF2BP2 antibody (Bethyl, USA) or normal IgG at 4 °C overnight. The beads were washed with Low-salt Wash buffer, High-salt Wash buffer and 1× PNK Buffer. The beads were resuspended in elution buffer and then divided into two groups, one group for the RNA isolation from IGF2BP2-RNA complexes and another group for the western blot assay of IGF2BP2.

**RNA immunoprecipitation (RIP) assay:** RNA immunoprecipitation was performed as previously described ^[17]^. RIP was analyzed with a Magna RIP RNA-Binding Protein Immunoprecipitation Kit (Millipore) according to the manufacturer’s instructions. RIP lysis buffer was used to lyse the cells, and magnetic beads coated with 5 µg of specific antibodies against mouse immunoglobulin G (Millipore) or IGF2BP2 (Bethyl) were incubated with prepared cell lysates overnight at 4 °C. All samples were incubated with proteinase K buffer at 55 °C for 30 min to separate the proteins after washing the RNA–protein complexes with RIP buffer six times. RNA was finally extracted by phenol–chloroform RNA extraction methods. Following centrifugation at 13000 rpm for 15 min at 4 °C, the supernatant was carefully removed, and the pellets were dried in air and then resuspended in 10 µL of RNase-free water. The relative interaction between Igf2bp2 and Notch1 transcripts was determined by qPCR and normalized to the input.

**RNA stability assay:** To analyze the objective RNA stability, Jurkat cells transfected with IGF2BP2 siRNA or control siRNA were plated in 12-well plates, treated with 5 µg/mL actinomycin D (Med ChemExpress) and then collected at the indicated time points. The total RNA was extracted by an EASYspin Plus kit (Yishan, China) and analyzed by RT–PCR. The mRNA half-life time was estimated according to a linear regression analysis.

**RNA pull down assay:** Single-stranded RNA containing methylated or unmethylated adenosine was synthesized by Biosune Biotechnology (Shanghai) Company. *NOTCH1* RNA was desthiobiotin-labelled by using a Pierce RNA 3′ End Desthiobiotinylation Kit (20163, Thermo Scientific), and RNA pull-down assays were carried out as described by the Pierce Magnetic RNA–Protein Pull Down Kit (20164, Thermo Scientific). Briefly, up to 50 pmol of RNA were denatured at 85 °C for 5 min and biotin-labelled by T4 RNA ligase. Then, biotin-labelled nucleic acid was incubated with 50 µL of streptavidin beads and 2 mg of Jurkat cell protein lysates. Finally, the eluted RNA-binding protein complexes were boiled and assayed with an anti-IGF2BP2 antibody.

**Human T-ALL xenograft:** The animal studies were conducted in compliance with institutional guidelines and were approved by the Medical Ethics Committee of Qilu Hospital of Shandong University. Jurkat cell xenografts were generated as previously described. The six-week-old male M-NSG mice (Model Animal Research Center of Nangjing University) were irradiated at 1 Gy before a tail vein injection of 4×10^6^ Jurkat cells infected with inducible LV2-m IGF2BP2-KD or LCV2-m Control virus. Weekly monitoring of the mice for circulating leukemia cells in peripheral blood was performed by an analysis of human CD45 expression with flow cytometry. The mice were sacrificed when they demonstrated characteristic disease symptoms or became moribund. The two groups of mice were sacrificed at the same time point. The cells were then isolated from the spleens by mechanical disaggregation and red blood cell lysis, and collected from bone marrow by flushing the femurs with PBS. The human CD45 surface marker was assessed by a flow cytometry analysis of the leukemia burden in vivo. Spleen sectionsand bones were prepared for immunohistochemistry staining. For JX5 treatment study in vivo, the six-week-old male M-NSG mice were irradiated at 1 Gy before tail vein injection of 5×10^6^ Jurkat cells. One week later, daily treatment with vehicle or JX5 (1 mg/kg or 2 mg/kg) by intraperitoneal injection (n=5). Leukemia burden was evaluated by assessing the degree of organomegaly and T-ALL cell infiltration. The mice body weight, pellets, and the structures of small intestine, colon and stomach were monitored to evaluate the compound’s gastrointestinal toxicity.

**Fluorescence quenching assay:** The interaction between the IGF2BP2 recombinant proteins and hit compounds was determined by fluorescence titration as previously described. IGF2BP2 proteins were dialyzed against PBS and incubated with different concentrations of hit compounds. The fluorescent quenching was conducted at 25 °C and the fluorescence spectra were recorded by a Cytation5 Multi-Mode Plate Reader (BioTek) using excitation at 280 nm and emission at 332 nm. The dissociation constant (K_d_) of JX5 to IGF2BP2 was calculated as previously described.

**Statistical Analysis:** The statistical significance of the differences in groups was determined by using GraphPad Prism 8.0 (GraphPad Software). All data and error bars are presented as the mean ± SD (standard deviation) based on experiments performed at least in triplicate. The data points were gathered such that significant variations could be observed in the data. Differences between two groups were analyzed using an unpaired Student’s t test. A Mann-Whitney U test was used for cases with unequal variances. Survival was presented with a Kaplan-Meier survival plot. P < 0.05 was considered statistically significant.

**Supplementary figures and figure legends**

**
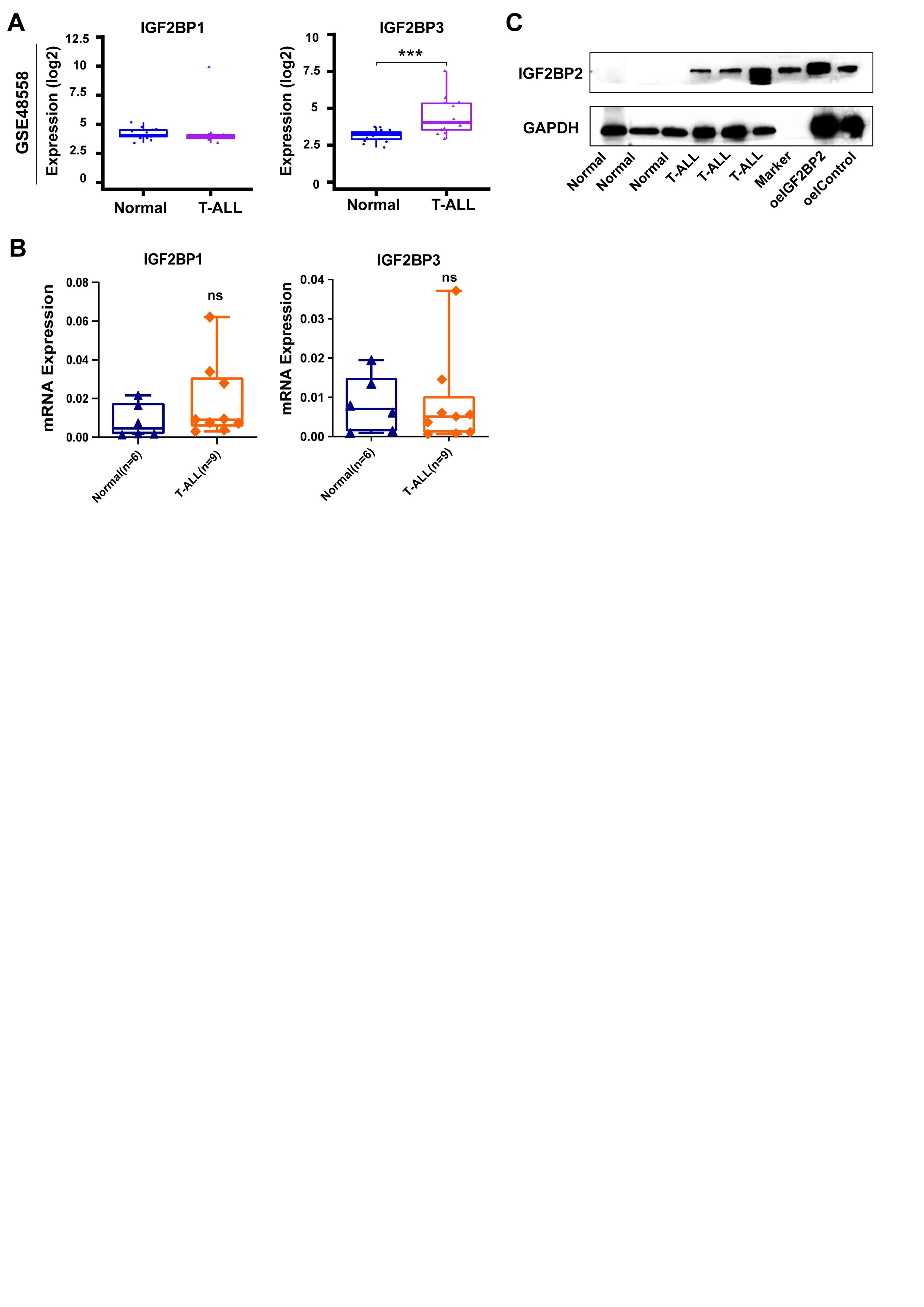
**

**Supplementary figure 1 Expressions of IGF2BPs in T-ALL samples and cell lines. A)** IGF2BP1/3 expression levels in normal T cells (n=17) and primary T-ALL patients cells (n=13) from GEO database, GSE48558. **B)** IGF2BP1/3 mRNA expression levels were analyzed in normal human bone marrow cells (n=6) and primary T-ALL patients cells (n=9) by RT-qPCR assays. **C)** IGF2BP2 protein expression level was assessed in normal human bone marrow cells (n=3), primary T-ALL patients cells (n=3) and oeIGF2BP2 Jurkat cells as positive control by western blot; the marker band indicates 70 kDa. Data are mean ± SD values. * P < 0.05; ** P < 0.01; *** P < 0.001.

**
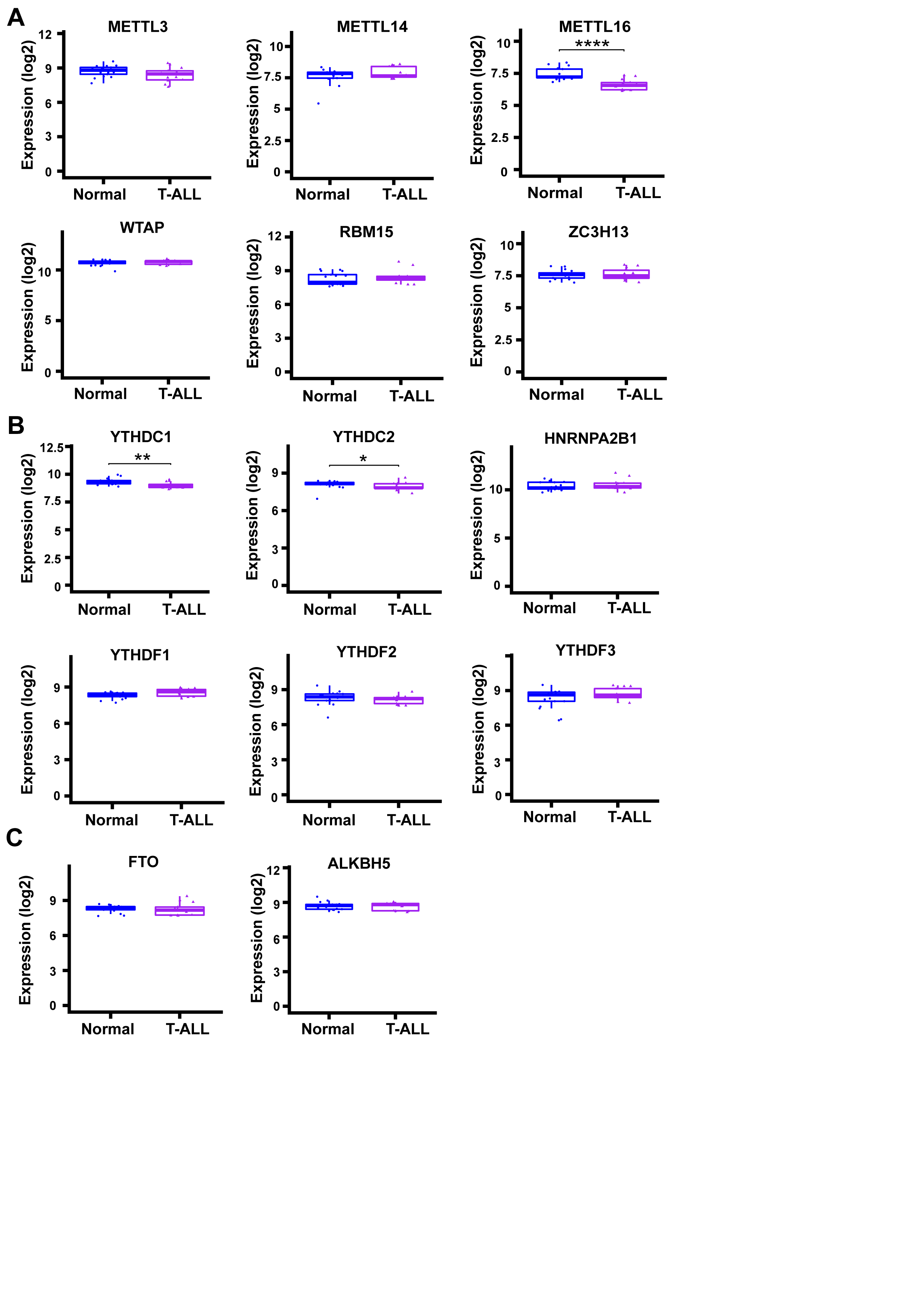
**

**Supplementary figure 2 Expressions of m^6^A modification enzymes in T-ALL.**

1. The expression levels of “writer” genes (METTL3, METTL14, METTL16, WTAP, RBM15, ZC3H13) in normal T cells (n=17) and primary T-ALL patients cells (n=13) from GEO database, GSE48558. **B)** The expression levels of “eraser” genes (YTHDC1, YTHDC2, HNRNPA2B1, UTHDF1, YTHDF2, YTHDF3) in normal T cells (n=17) and primary T-ALL patients cells (n=13) from GEO database, GSE48558. **C)** The expression levels of “reader” genes (FTO, ALKBH5) in normal T cells (n=17) and primary T-ALL patients cells (n=13) from GEO database, GSE48558. Data are mean ± SD values. * P < 0.05; ** P < 0.01; *** P < 0.001.

**
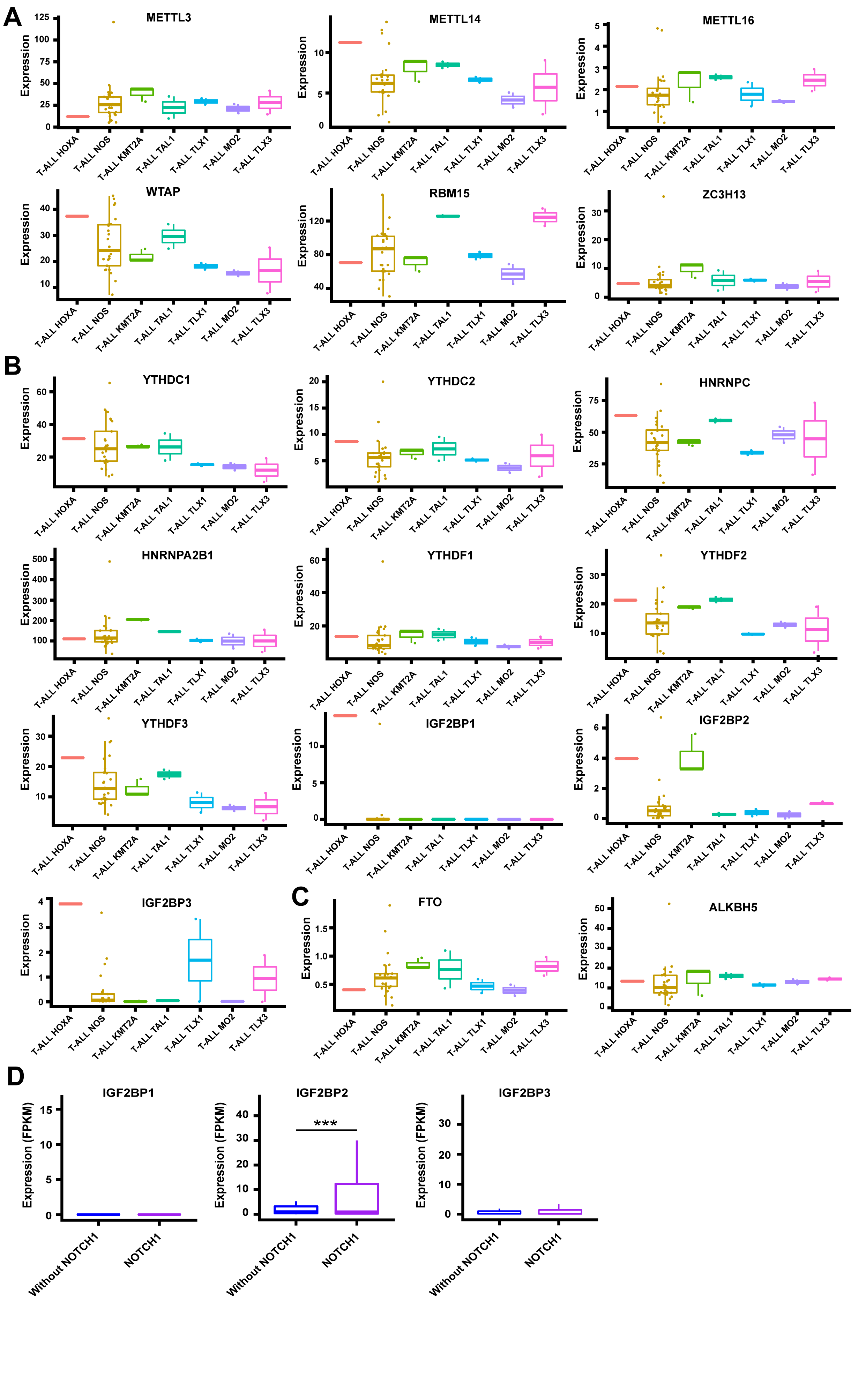
**

**Supplementary figure 3 Expressions of m^6^A modification enzymes in different T-ALL subtypes. A)** The expression levels of “writer” genes (METTL3, METTL14, METTL16, WTAP, RBM15, ZC3H13) in different T-ALL subtypes from PECAN database. **B)** The expression levels of “eraser” genes (YTHDC1, YTHDC2, HNRNPA2B1, UTHDF1, YTHDF2, YTHDF3, IGF2BPs) in different T-ALL subtypes from PECAN database. **C)** The expression levels of “reader” genes (FTO, ALKBH5) in different T-ALL subtypes from PECAN database. RNA-Seq data sets of human T-ALL are classified into subgroups with the following genes: HOXA (n = 1), NOS, not otherwise specified (n = 25), KMT2A (n = 3), TAL1 (n = 2), TLX1 (n = 2), LMO2 (n = 2), and TLX3 (n = 2). **D)** The expression levels of IGF2BPs in T-ALL NOTCH1 mutation subtypes from NCI TARGET database (phs000464). RNA-Seq data sets of human T-ALL are classified into subgroups with the following genes: NOTCH1 mutation (n = 187), without NOTCH1 mutation (n = 77). Data are mean ± SD values. * P < 0.05; ** P < 0.01; *** P < 0.001.

**
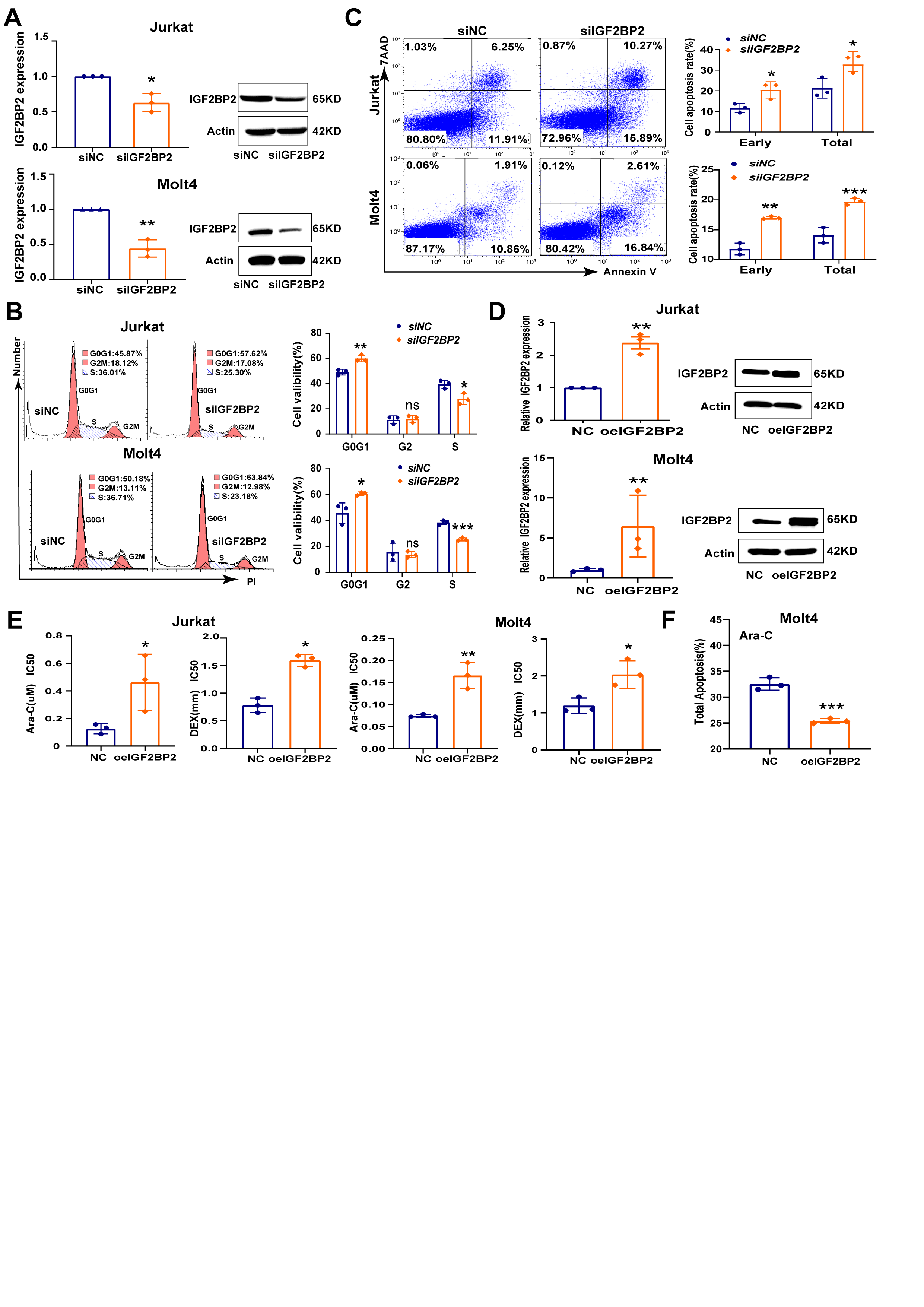
**

**Supplementary figure 4 IGF2BP2 promotes cell survival and impairs chemotherapy sensitivity in T-ALL. A)** Jurkat and Molt4 cells were transfected with IGF2BP2 siRNA (siIGF2BP2) or control siRNA (siNC). IGF2BP2 mRNA expression levels (n=3) and protein expression levels were analyzed by RT-qPCR and western blots. **B)** Cell cycle analysis using flow cytometry after staining with propidium iodide (PI), and the phase distribution was quantified with model fitting using ModFit (left). Percentages were representative of cell cycle distributions from three replicate experiments in Jurkat cells (siIGF2BP2 or siNC) and Molt4 cells (siIGF2BP2 or siNC) (right) (n=3). **C)** Apoptosis analysis of Jurkat cells (siIGF2BP2 or siNC) and Molt4 cells (siIGF2BP2 or siNC) were measured by flow cytometry (left). Percentages were representative of cell apoptosis from three replicate experiments (right) (n=3). **D)** Jurkat and Molt4 cells were transduced with IGF2BP2 lentivirus (oeIGF2BP2) or control lentivirus (NC). IGF2BP2 mRNA expression levels (n=3) and protein expression levels were analyzed by qPCR and western blots. **E)** IC50 values of Jurkat cells (oeIGF2BP2 or NC) and Molt4 cells (oeIGF2BP2 or NC) were calculated according to cell growth inhibition measured by CCK8 assay after 48 h treatment with serial dilutions of Ara-C and Dexamethasone (n=3). **F)** Apoptosis analysis of Molt4 cells (oeIGF2BP2 or NC) after 24h treatment with Ara-C was measured by flow cytometry. Percentages were representative of cell apoptosis from three replicate experiments (n=3). Data are mean ± SD values. * P < 0.05; ** P < 0.01; *** P < 0.001.

**
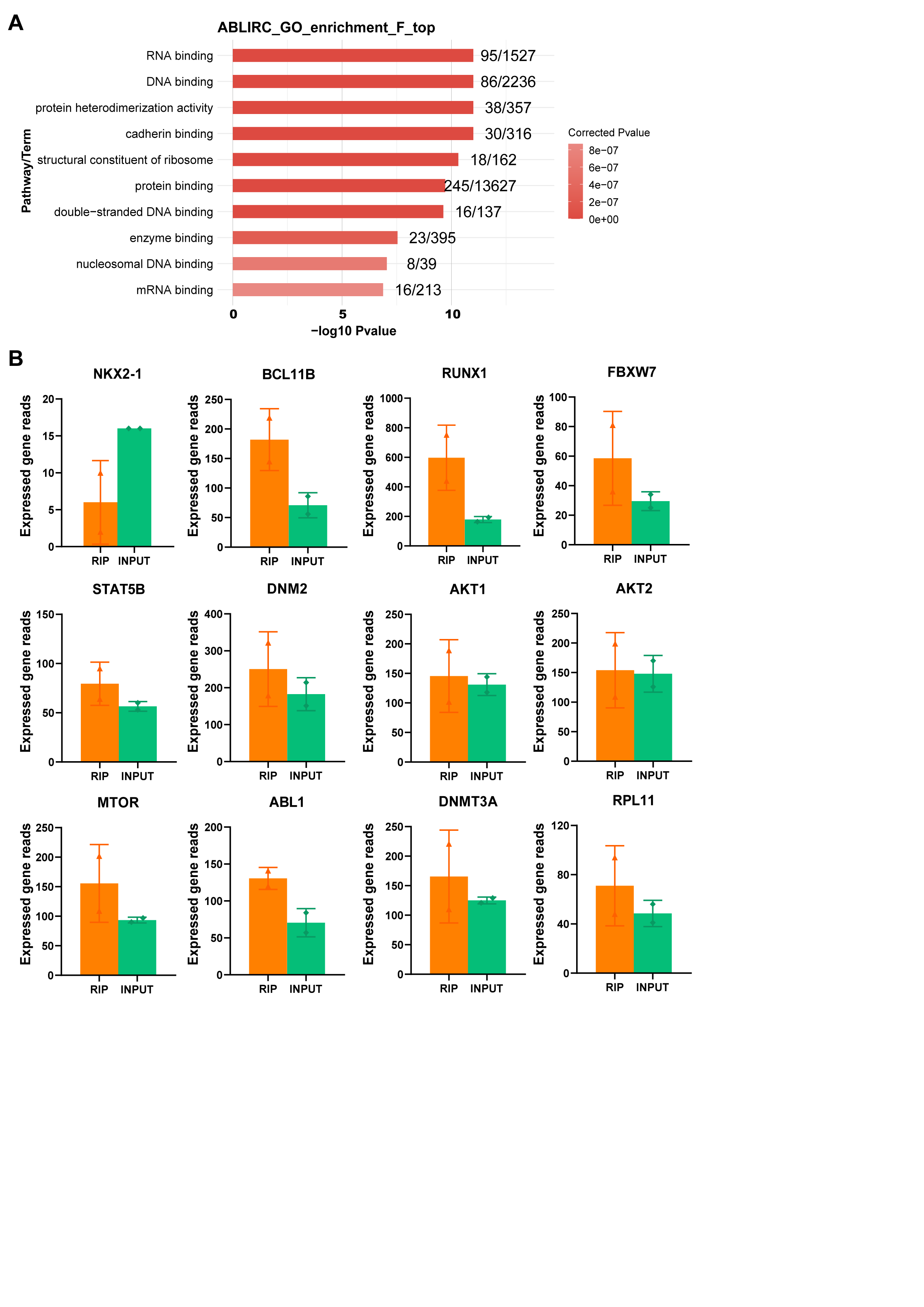
**

**Supplementary figure 5 iRIP-sequence of IGF2BP2 in T-ALL cells.**

1. The top 10 enriched GO biological processes of the IGF2BP2-bound genes.
2. The expression reads levels of gene (NKX2-1, BCL11B, RUNX1, FBXW7, STAT5B, DNM2, AKT1, AKT2, MTOR, ABL1, DNMT3A) in IGF2BP2 binding iRIP-sequence and Input control from two replicate experiments.

**
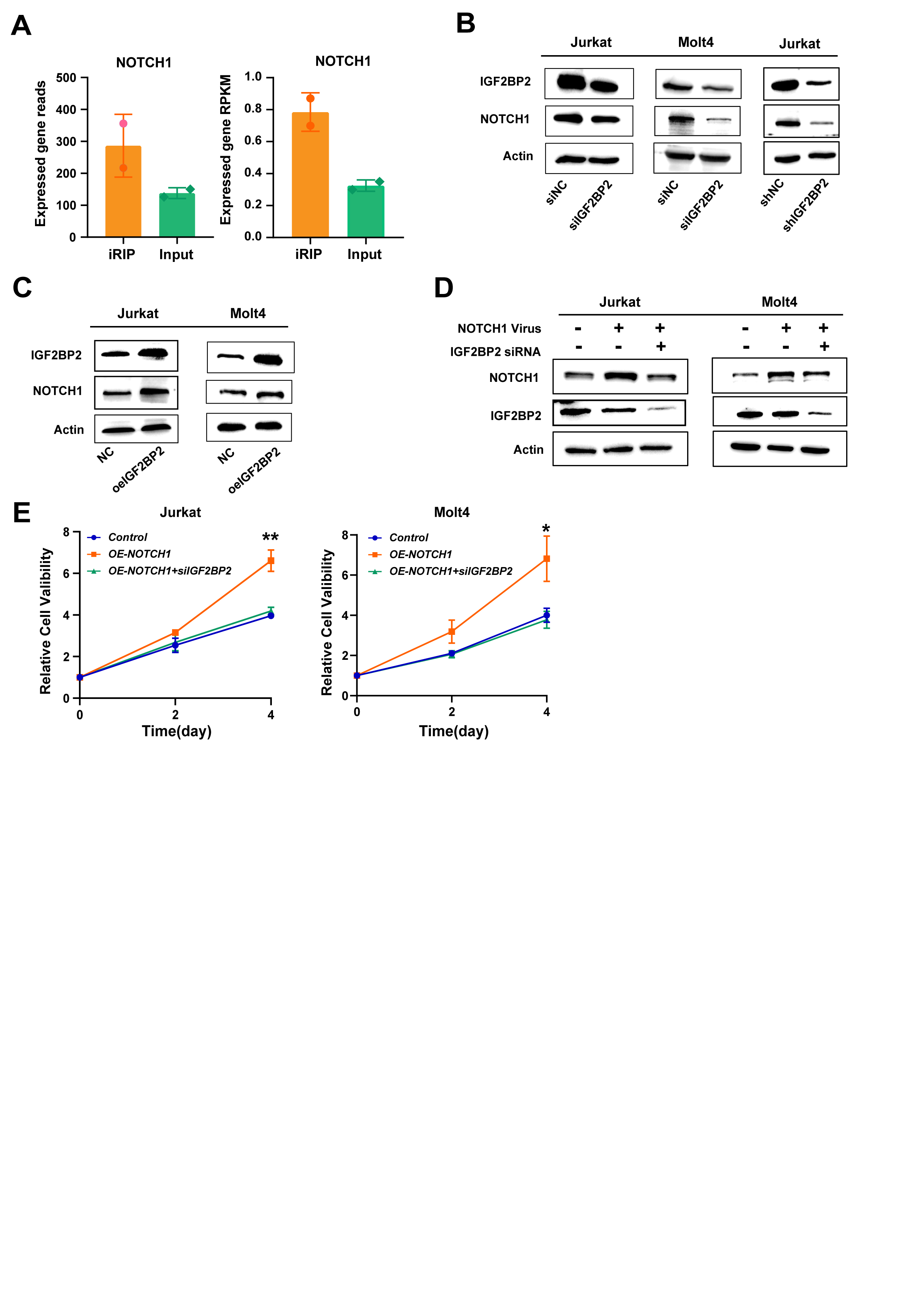
**

**Supplementary figure 6 Transcriptome-wide iRIP-sequence assays identifies potential targets of IGF2BP2 in T-ALL. A)** The expression levels of NOTCH1 reads (left) and RPKM (right) in IGF2BP2 binding iRIP-sequence and Input control from two replicate experiments (n=2). **B)** IGF2BP2 and NOTCH1 protein expression levels of Jurkat cells (siIGF2BP2 or siNC), Molt4 cells (siIGF2BP2 or siNC) and Jurkat cells (shIGF2BP2 or shNC) were analyzed by western blot. **C)** IGF2BP2 and NOTCH1 protein expression levels of Jurkat cells (oeIGF2BP2 or NC) and Molt4 cells (oeIGF2BP2 or NC) were measured by western blot. **D), E)** Jurkat and Molt4 cells, transduced with NOTCH1 or control virus, were treated with IGF2BP2 siRNA for 72h. IGF2BP2 and NOTCH1 protein expression levels were analyzed by western blot (D), meanwhile, cell proliferation was measured by CCK8 assay (E) (n=3). Data are mean ± SD values. * P < 0.05; ** P < 0.01; *** P < 0.001.

**
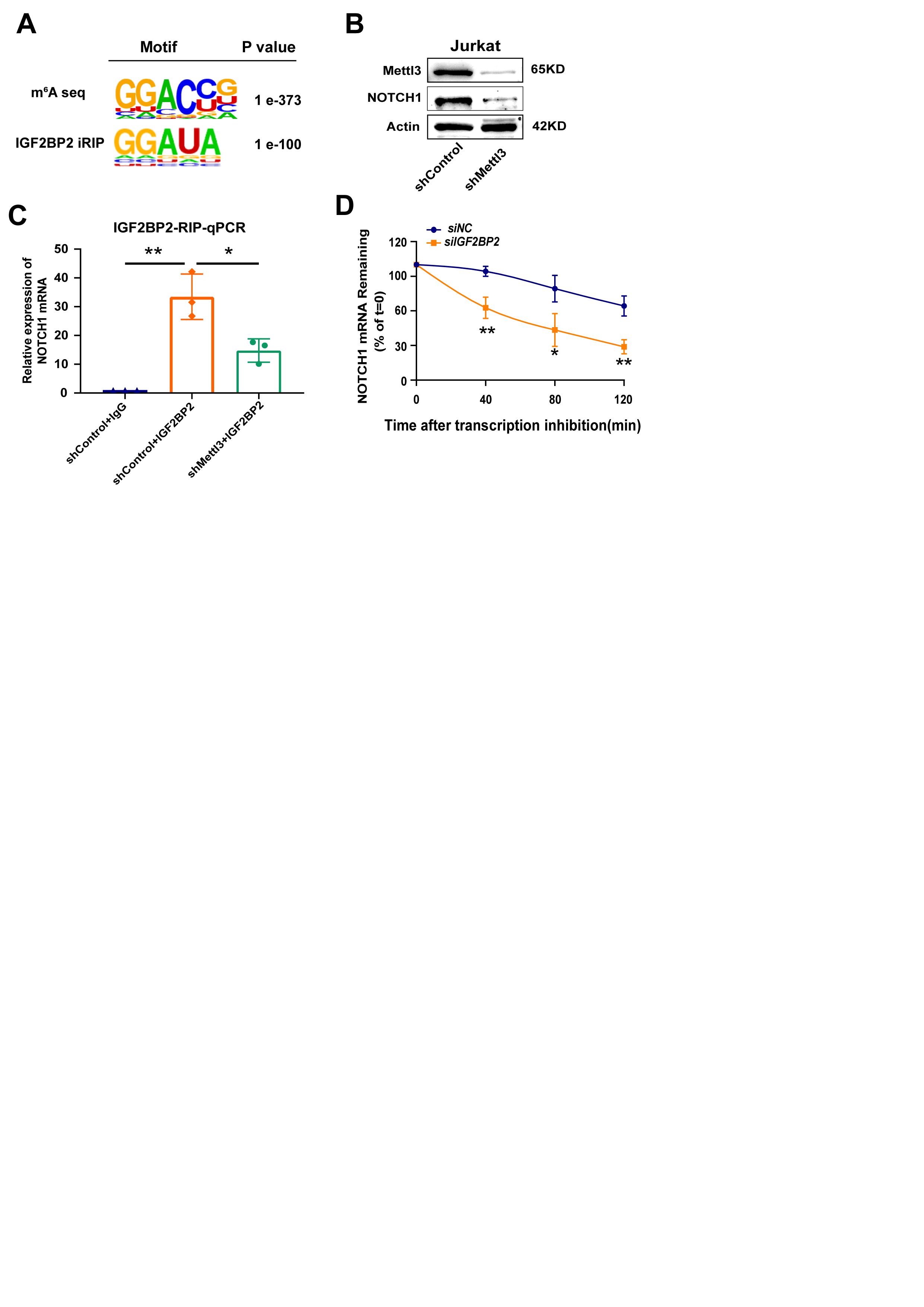
**

**Supplementary figure 7 IGF2BP2 enhances NOTCH1 mRNA stability via an m^6^A-dependent manner. A)** Top consensus sequences of IGF2BP2-binding motif from IGF2BP2-RIP data and m^6^A-modification motif detected by m^6^A-sequence. **B)** METTL3 and NOTCH1 protein expression level were analyzed by western blot in Jurkat cells，which were transduced with METTL3 knockdown virus and control virus. **C)** The enrichment of NOTCH1 mRNA was assessed by IGF2BP2-RIP-qPCR in Jurkat cells, which were transduced with Mettl3 knockdown virus and control virus. Results are presented relative to those obtained with immunoglobulin G (IgG) (n=3). **D)** IGF2BP2 mRNAs degradation were detected by RT-qPCR in Jurkat cells, which were treated with actinomycin D for the indicated times. The residual RNAs were normalized to 0 h (n=3). Data are mean ± SD values. * P < 0.05; ** P < 0.01; *** P < 0.001.

**
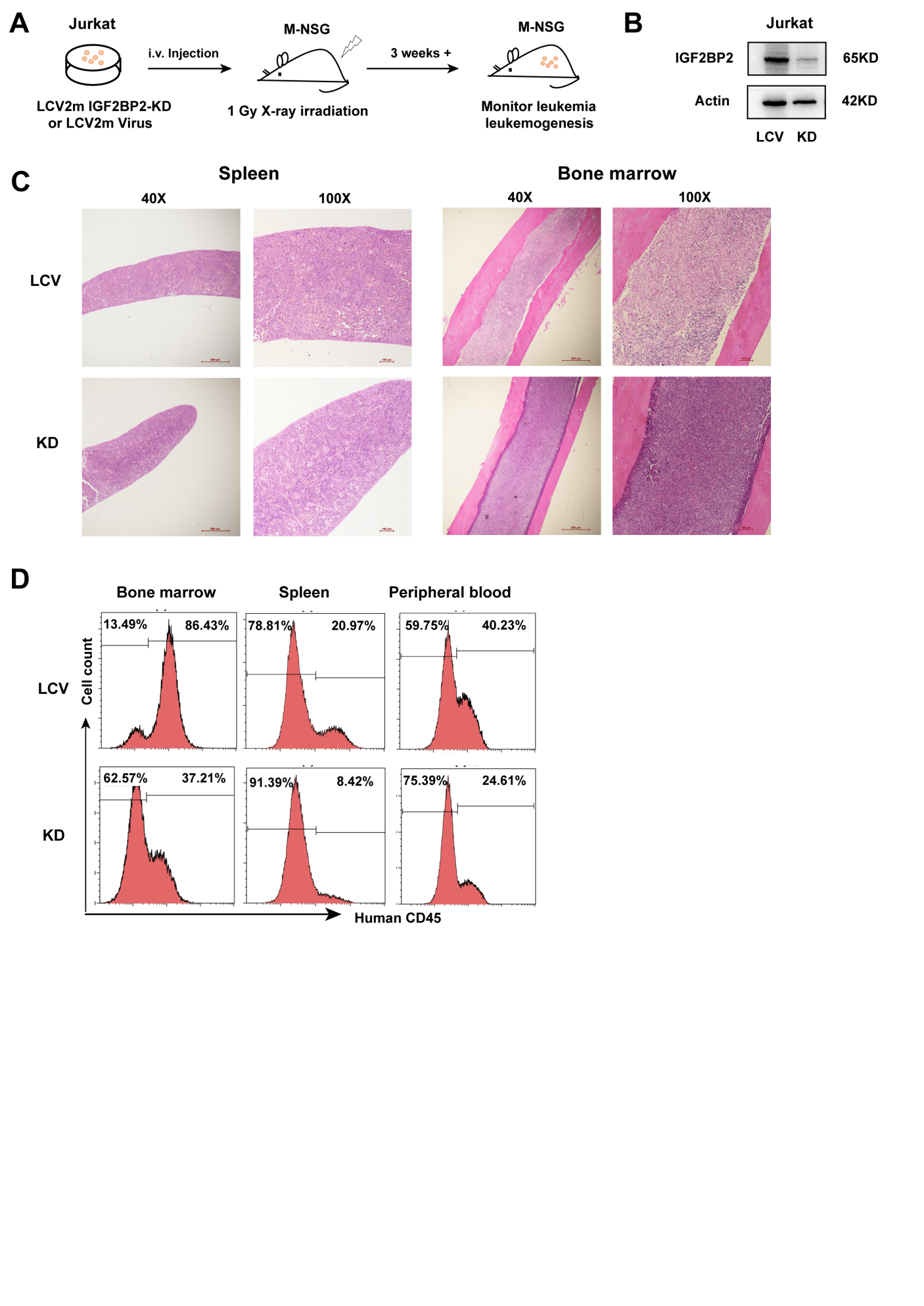
**

**Supplementary figure 8 IGF2BP2 knockdown significantly decreases leukemia burden in the human T-ALL xenograft. A)** Schematic representation of human T-ALL xenograft. Five million Jurkat cells, transduced with LCV2m IGF2BP2-KD virus and LCV2m control virus, were injected into irradiated M-NSG mice (1 gray). **B)** IGF2BP2 protein expression level was analyzed by western blot in Jurkat cells transduced with LCV2m IGF2BP2-KD virus and LCV2m control virus. **C)** Representative Hematoxylin and eosin (H&E) images (original magnification 40× and 100×) from two groups showing different infiltration levels of tumour in spleen and bone marrow. **D)** Human CD45^+^ cells from bone marrow, spleen and peripheral blood were analyzed by flow cytometry.

**
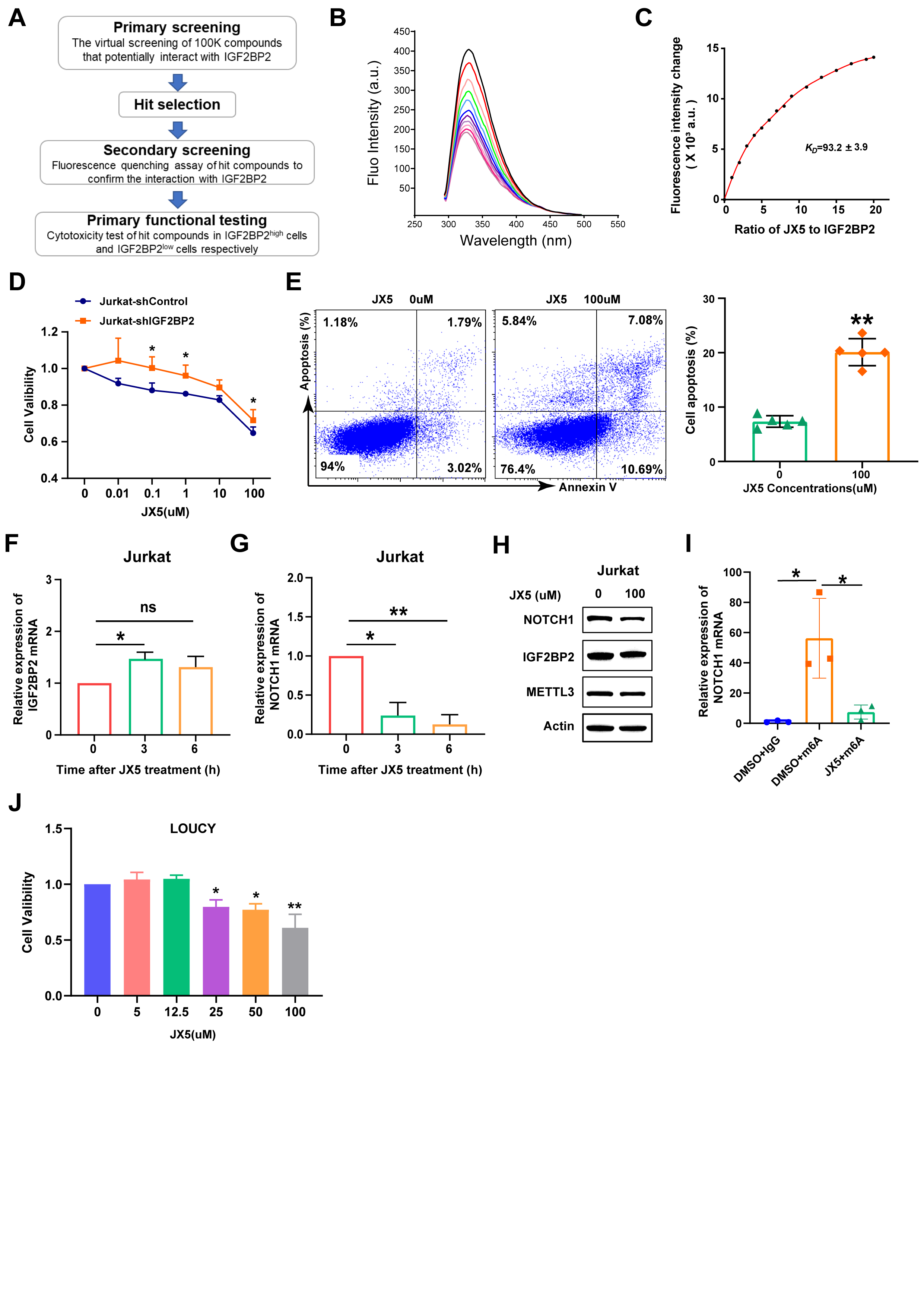
**

**Supplementary figure 9 Small-molecule inhibition of IGF2BP2 as a strategy against T-ALL. A)** Screening strategy of IGF2BP2 small molecule inhibitors. **B)** The change in fluorescence intensity of IGF2BP2 upon increasing concentration of JX5. The lines indicated the titration of intensity of IGF2BP2 (10 μM) with JX5 against IGF2BP2. Compound concentrations were 0, 10, 30, 50, 70, 90, 110, 130, 150, 170 and 200 μM from up to down, respectively. **C)** The change in fluorescence intensity of IGF2BP2 upon increasing concentration of JX5. The plot indicated the titration of intensity of IGF2BP2 (10 μM) with JX5 against IGF2BP2 single at λ_em_ = 332 nm. compound concentrations were 10, 20, 30, 40, 50, 60, 70, 80, 90, 110, 130, 150, 170, 190 and 200 μM, respectively. The dissociation constant (K_d_) is obtained from three independent experiments and shown as mean ± SD. **D)** Cytotoxicity testing of JX5 in the Jurkat cells (shControl and shIGF2BP2) (n=3). **E)** Apoptosis analysis of Jurkat cells were measured by flow cytometry after 72 h treatment with JX5. Percentages were representative of cell apoptosis from three replicate experiments (n=5). **F), G)** IGF2BP2 (F) and NOTCH1 (G) mRNA expression levels of Jurkat cells were measured by RT-qPCR after 3h or 6h treatment with JX5 (n=3). **H)** NOTCH1, IGF2BP2 and Mettl3 protein expression levels of Jurkat cells were measured by western blot after 72h treatment with JX5. **I)** The m^6^A levels of NOTCH1 was measured by m^6^A RIP-qPCR in JX5 treated Jurkat cells (n=3). **J)** Cytotoxicity testing of JX5 in the LOUCY cells (n=3). Data are mean ± SD values. * P < 0.05; ** P < 0.01; *** P < 0.001.

**
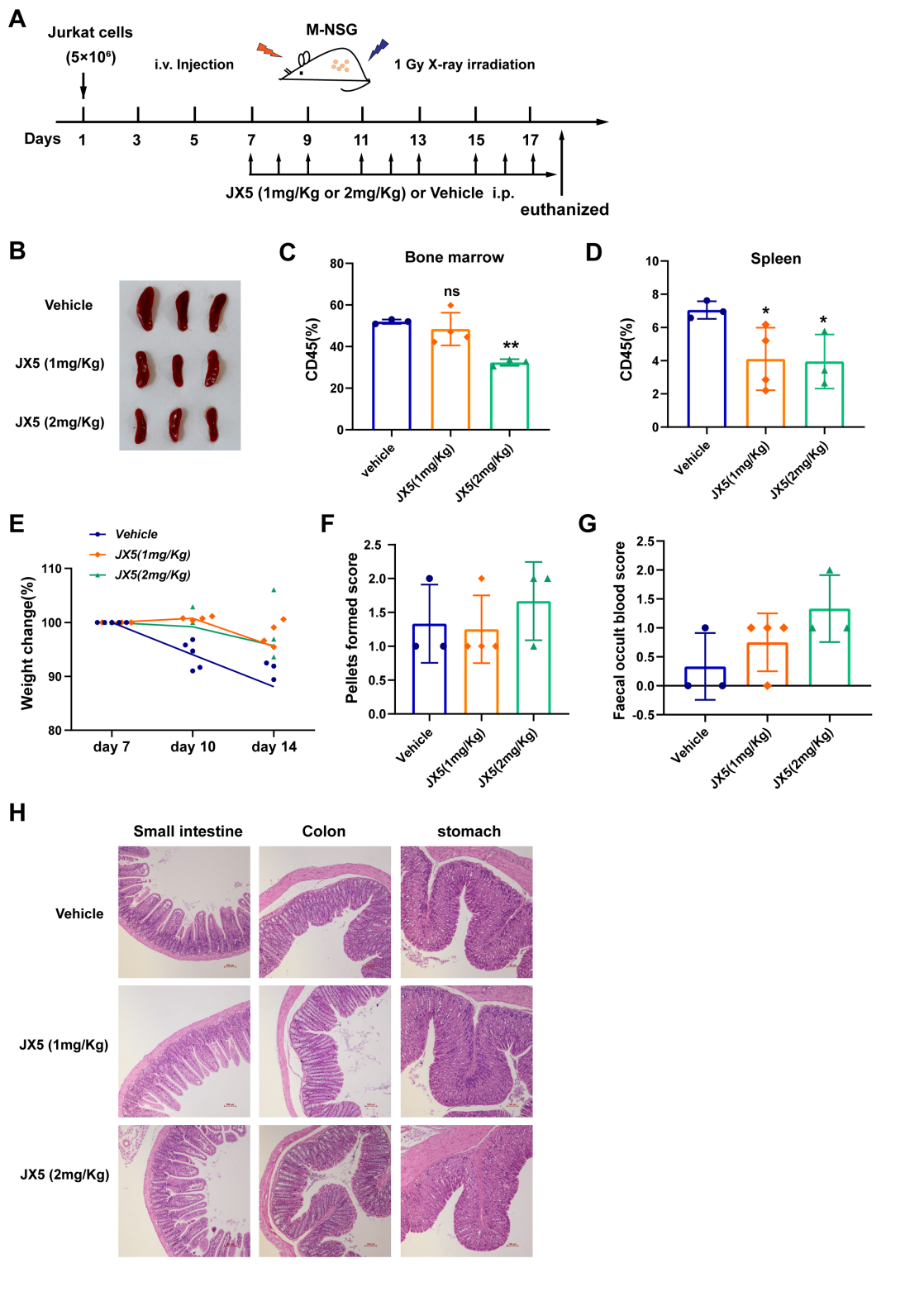
**

**Supplementary figure 10 JX5 prevents T-ALL expansion in vivo. A)** Schematic representation graph of the therapy of JX5 in T-ALL xenograft mice model. Five million Jurkat cells were injected into irradiated M-NSG mice (1 gray irradiation), followed by treatment with vehicle or JX5(1 mg/kg or 2 mg/kg). **B)** Representative images of mice spleen treated with vehicle or JX5(1 mg/kg or 2 mg/kg). **C) and D)** Human CD45^+^ cells from bone marrow and spleen were analyzed by flow cytometry. **E)** Body weight changes of three groups were monitored at day 7, day 10 and day 14. **F)** Pellets formed score of mice treated with vehicle or JX5 (1 mg/kg or 2 mg/kg). **G)** Faecal occult blood score of mice treated with vehicle or JX5 (1 mg/kg or 2 mg/kg). **H)** Representative images of hematoxylin and eosin (H&E) stained small intestine, colon and stomach of mice treated with vehicle or JX5 (1 mg/kg or 2 mg/kg). Scale bar 100 μm. (representative images of n=3-4)

**Supplementary file 1** The overlap genes.

| **GENES** | **GENES** | **GENES** | **GENES** | **GENES** | **GENES** |
| --- | --- | --- | --- | --- | --- |
| TET3 | SRSF4 | AFF1 | ZNF843 | PROC | TCP11L2 |
| ANKS6 | TRIO | SLC9A8 | NUDT16 | PPP1R35 | BCL7A |
| ALKBH4 | NCKAP1L | CYP2A6 | PQLC1 | CCDC28B | SLC22A14 |
| TRAM2 | APBA2 | RABGAP1 | CLMN | GOLGA6L10 | SH3GLB2 |
| KDM4B | TXNDC12 | GTF2E1 | DCP1B | PFKL | VPS26B |
| GBA2 | ZGPAT | PI4KA | HIP1R | PPIL2 | MAP4K1 |
| TSPAN9 | DTYMK | MIIP | DAZAP1 | EXOSC5 | KCTD13 |
| DNAAF5 | ZNF516 | CENPT | AARS | ACADVL | CLN8 |
| PALD1 | CAMKMT | PHF2 | LZTS2 | RALGPS1 | CREB3L2 |
| RBMS2 | UFSP2 | IFT43 | C16orf58 | PPFIA4 | RECK |
| C11orf49 | BSN | MSLNL | ST3GAL6 | KIAA1958 | DENND1A |
| EPN1 | STAT5B | SLC38A10 | PPIA | LUC7L | ZNF444 |
| CDK2AP1 | CORO1B | ACYP2 | ARFRP1 | ARPC1B | WIPI2 |
| ZXDA | HDAC6 | RPS2 | AGRN | ARHGAP35 | R3HDM1 |
| TTLL3 | FLNA | NEU3 | TRAK1 | TCEA2 | FAM193B |
| PRKRIP1 | CC2D1A | MVD | CYTH1 | NDUFB11 | SLC9A1 |
| CLSTN3 | CELSR1 | NKX2-1 | PPARGC1B | TMEM184B | PIDD1 |
| KIAA1147 | NCKAP5L | ZFYVE28 | BTNL9 | ACOT8 | NAV1 |
| RCOR1 | STAT2 | DAGLA | SUFU | STIM1 | NFIC |
| GHDC | RAB40B | MYH9 | PPL | ACTR1A | ADARB1 |
| ADAMTS10 | AXIN1 | SLC35F4 | LCK | ZNF71 | LGR4 |
| TWF2 | PARP10 | RANBP10 | RPLP2 | ZNF445 | MKRN1 |
| AIFM3 | MSL1 | HIST1H3I | BABAM1 | LGR6 | FAAP20 |
| RNF44 | TUBG1 | DOCK3 | CDK16 | GNAL | SLC6A8 |
| TRIM52 | ZBTB16 | RHD | ANO9 | HOMER3 | SLC25A44 |
| CLEC16A | U2AF2 | PGAM1 | WNK2 | H1F0 | DHX30 |
| MAP2K3 | FOXK2 | TRAPPC9 | CCDC136 | PEX11G | IGLL1 |
| RPL37A | SEC16A | TRIM8 | FAT2 | GPR107 | ATG16L1 |
| MTMR3 | HR | TRIM27 | CSNK1E | SSBP3 | DDX49 |
| KCNE1 | AFF2 | CYFIP1 | SATB2 | STK36 | DUS1L |
| SNRPG | FMNL3 | HEXDC | SKIV2L | RPAIN | TFPT |
| TPPP | SH3GL1 | SETD1A | ZNF518B | NBPF19 | EZH1 |
| PITPNM3 | ADAM17 | CHD6 | UBL5 | ZBTB7B | KMT2C |
| LSAMP | SRCAP | SLC4A2 | DDX54 | MYO5B | SNX1 |
| IRAK1 | ERICH1 | TECPR2 | LIN52 | PPP1R16A | UBAC1 |
| TULP1 | ZNF8 | INPP5E | IFT140 | TP53I11 | MBD5 |
| HDAC7 | TBRG4 | LIN7B | VAV2 | NDUFB7 | SIVA1 |
| RNF187 | ZNF142 | ABL1 | MED24 | UBE4B | JAKMIP1 |
| S100Z | C2CD2 | NAT10 | UBE2M | XAB2 | EP400 |
| GFOD1 | SRM | DPP6 | GGT7 | STARD9 | NOTCH2 |
| GALNT10 | SARM1 | CHCHD3 | APC2 | SMYD3 | VEGFB |
| DUSP7 | MAPK8IP3 | ZNF740 | AGO1 | UBTF | KY |
| ATP6AP1 | ZBBX | XRCC3 | PPP1R9B | WASF3 | ZNF16 |
| GIGYF2 | CDK18 | HMG20B | THRA | HGD | LGALS9 |
| DPF1 | C1orf35 | ATP13A1 | SERINC5 | CYB5R3 | VPS39 |
| ZNF710 | PLXND1 | DNM2 | CAD | SRRM2 | BFAR |
| RPL38 | CDH4 | DBN1 | VDAC1 | ZSCAN29 | PHACTR1 |
| TCAF2 | SEC61A1 | CRY2 | GOLGA2 | RNF10 | SMARCD1 |
| C14orf178 | PFDN2 | TTC17 | TMEM63B | MADD | P2RY13 |
| EPHB1 | C7orf50 | ANO8 | LRRC45 | KSR2 | GOLGA1 |
| UBAP2L | LPCAT1 | CERS4 | HK1 | TSNARE1 | PHF8 |
| LRCH4 | WDR88 | TPD52L2 | RPL37 | WNT8B | SAMD4B |
| TYMP | CEP131 | TANC1 | OS9 | RNF150 | DIS3L2 |
| SPEN | ANKH | SURF4 | SLC26A6 | ATP2A3 | RBM6 |
| PHC1 | KLC4 | COL11A2 | EML6 | TTYH3 | UNC13D |
| LARP1 | TTC28 | SUN1 | SSBP4 | VPS51 | SREBF2 |
| LRPAP1 | RELB | GUCD1 | ZADH2 | FBF1 | IGSF9B |
| HIST1H4I | RNF34 | MSS51 | PTPRS | CHST11 | MAST3 |
| CIRBP | KHNYN | CASK | CPT1B | TEX2 | UVSSA |
| NCOR2 | CBL | DGCR2 | LRP8 | NPLOC4 | FAM3A |
| ECE1 | DALRD3 | KDM6B | USP49 | AACS | SUGP2 |
| TRABD | RBM19 | DTNB | LEPR | KIF21B | CNOT3 |
| CSNK1G2 | RPL8 | CACNA1C | CSRNP3 | CCAR2 | NTHL1 |
| GP1BA | KCNC1 | DLST | SIN3B | PDE1B | GXYLT2 |
| PHF21B | SPTBN1 | HIPK1 | FCGBP | RXRB | TFB1M |
| FAM189B | EIF5AL1 | POLRMT | APEH | CCDC22 | D2HGDH |
| PRKCA | SGSM2 | AGO2 | PPFIA3 | SYVN1 | SLC35C2 |
| ZER1 | PLXNA1 | WDR5 | ELAVL3 | FYCO1 | PFN1 |
| OSBPL5 | MAP3K6 | PIH1D1 | MARCKSL1 | PHKA1 | KIAA2013 |
| POMGNT1 | MPRIP | NISCH | DICER1 | SMPD4 | FUBP3 |
| NR2C2 | LGALS3BP | EGLN1 | ERN1 | ATG16L2 | VPS37B |
| TOP1MT | GABBR1 | SERPINA10 | ORAI1 | NEURL4 | CCDC12 |
| ICAM3 | PLD2 | PLEKHG2 | RUVBL2 | TBC1D20 | CLASP1 |
| MLLT1 | BAG6 | AGPAT3 | ZNF346 | NCAPD3 | DNAJA3 |
| ENO2 | SCAF4 | ZHX3 | IGFL4 | STARD3 | RERE |
| DLG5 | MAP3K10 | XPO7 | DISC1 | ZZEF1 | IKZF3 |
| BRD4 | C1orf226 | UBE2I | PHF13 | PPP1R3B | MICALL2 |
| TNKS1BP1 | ZNF333 | RNASEH2C | TONSL | TRAF3 | AP2A1 |
| AUTS2 | PLEC | HGS | CNNM2 | CHD2 | PLEKHM1 |
| NFX1 | REXO1 | BCL11B | LDLR | TXNRD2 | ZBTB17 |
| TCF7 | GPSM1 | PPFIBP2 | TAF1C | FGFRL1 | SRSF5 |
| ZNF276 | TMEM243 | ABLIM2 | PACS2 | TNFRSF10B | PHF7 |
| PPM1L | PROSER3 | PODXL | DGKD | FBRSL1 | PLEKHF1 |
| SDCBP2 | CPNE1 | MAN2A2 | POM121 | LONP2 | POR |
| EIF3G | PRR5L | APMAP | THOC3 | TNFRSF14 | FBRS |
| MMP25 | STX4 | PRPF3 | MTCH1 | BSCL2 | USP10 |
| CMTM4 | ADTRP | SMARCD2 | CD81 | HYAL2 | ATXN2L |
| SLC12A7 | B4GALNT3 | MEGF8 | ARID3A | STAT3 | TNNT3 |
| DPP3 | CCDC33 | CAPN15 | ZBTB8A | SGTA | LUC7L3 |
| BAIAP3 | NME4 | SLA | BRSK2 | EXOC8 | RNF144A |
| TNPO2 | CMIP | HECTD4 | KIAA1211 | LGMN | EIF4H |
| HDGF | COL26A1 | NFKBIL1 | RPL35A | PDE4DIP | EIF5A |
| ARHGEF1 | RAD54L2 | ZNF121 | MBD3 | RNH1 | P4HA1 |
| AGAP1 | NAV2 | SFT2D2 | ENTPD4 | SCAP | MTCL1 |
| PPP6R2 | KIAA1210 | ACTL10 | ATP6V1F | LRRC4 | MOV10 |
| WWOX | SEC24C | FAM102A | RIMKLA | MRPS26 | SBF1 |
| GBF1 | ZMIZ2 | ZNF517 | DEAF1 | MZT2A | FBXO42 |
| ABCB8 | FAM32A | CRKL | RBM5 | KIAA0513 | LASP1 |
| SUMF2 | KLC1 | MRPL52 | KDM4C | AQP3 | LRRC61 |
| PRPF18 | STAB2 | CLUH | CHSY1 | SMAP2 | SOX12 |
| AGAP3 | LTB4R | PPP1R37 | ATP2B2 | MYO10 | ZNF609 |
| HEATR5A | ZNF490 | AMER1 | QPCTL | MORN5 | TSHZ1 |
| CEP85 | UBA1 | SEC22A | GOLGA3 | TMEM164 | COA5 |
| TRPV1 | LRRC27 | ZNF429 | CSNK2B | PPIP5K1 | DHX8 |
| GAS6 | ERH | GUCY1A2 | WTIP | PLD3 | ALG3 |
| METTL3 | ITGAL | VPS9D1 | TTYH1 | TKT | CLTCL1 |
| MPST | LRRC14 | FAM210A | PIN1 | SLC37A2 | FNBP4 |
| ZNF573 | RCSD1 | HPS4 | RPS9 | ZC3H7B | PHF19 |
| SNTB2 | B3GALNT2 | PRR36 | CP | ELL | KLHL11 |
| MED25 | SLC41A1 | ITFG2 | TRRAP | SCAMP2 | CAPN1 |
| SLC25A45 | ZNF668 | OGFR | BCAS3 | ATP8B3 | FKBP15 |
| ZKSCAN1 | WIZ | MRPS5 | AMPD3 | EIF1B | ALDH18A1 |
| ALMS1 | NDUFV1 | ARID3B | PTPN6 | PSMD3 | MAML3 |
| SLC2A1 | HERC3 | NACC2 | LTBP1 | NFIX | ADGRB2 |
| SLC25A53 | USP7 | ZNF862 | SLC29A4 | F11R | NOTCH3 |
| CANT1 | POLR3D | ARHGAP1 | FAM129C | ARHGEF2 | SIRT4 |
| CCDC74B | RPS6KA1 | DBH | STK11IP | ROBO1 | BCL9L |
| DAB2IP | SAFB2 | SGSM3 | PPP1R12B | ERCC2 | ZNF7 |
| CDK9 | EWSR1 | C2orf15 | RPL18A | PHC3 | LMAN2 |
| CREBZF | GANAB | GTF3C1 | PRRC2B | COX14 | UBE2V1 |
| TSPAN14 | RNPEPL1 | ZFYVE27 | FAM120B | ADCY7 | PIAS4 |
| CDH26 | SRRM1 | CTDP1 | CACNB3 | CELF1 | PRKAR1B |
| SREBF1 | BTBD2 | CDH2 | SDHAF4 | MED16 | CNNM3 |
| NOMO2 | ZNF160 | KMT2D | TBC1D2B | PPIL6 | ZBTB40 |
| RABL6 | PBX2 | RORB | SIPA1L3 | TOM1L2 | FAAP100 |
| ATP1A3 | NF2 | MLC1 | PTBP1 | ING5 | MYLK |
| UBE2O | FOXK1 | MYO9B | NDST1 | MAX | PTK7 |
| TICRR | TUBGCP2 | NDUFA13 | IFT20 | ZNF496 | ACIN1 |
| TRAPPC12 | DDX24 | AKT2 | RNF157 | PSMB3 | RASA3 |
| KIF26A | NTSR1 | PEX13 | COX17 | UNC45A | CHAF1A |
| GPC1 | AAMDC | WDFY2 | NBPF12 | ZNF605 | SCD |
| MLXIP | TLE3 | ZNF512B | EMC10 | DCAF7 | HYOU1 |
| EYA3 | STK35 | SF3A1 | CREBBP | CPLX1 | KIFC3 |
| DVL2 | UNC13B | POLR2J | SLAMF1 | LZTR1 | GSDMD |
| TAF6 | CYP2W1 | MTFMT | SIDT2 | KCNQ3 | TRAF7 |
| CRELD1 | TCF7L1 | ATXN2 | ESYT2 | ASB16 | DHX16 |
| PTK2B | PTGR2 | HAUS5 | MGAT5B | ZNF362 | TRIM56 |
| GATAD2B | TMEM129 | ANKZF1 | ARVCF | DHX34 | NUP188 |
| GRIN2D | ZNF500 | ADGRG1 | CARS | EXOC7 | CACNG8 |
| C16orf74 | RNF165 | FBXL16 | IGFBP2 | SPAG9 | ZSCAN2 |
| CMYA5 | TSPO2 | RAB35 | MVB12B | IST1 | HEG1 |
| SPATS2L | PLCG1 | SIRT2 | ZNF343 | SLC25A37 | SLC35F2 |
| MFGE8 | ANKMY1 | FAM156A | SH3BP2 | SUMO3 | ACO2 |
| VCL | LETM1 | PTPRK | INCENP | AHCY | CEP250 |
| EMD | EBP | SS18L1 | IFI27L1 | TBC1D13 | THAP4 |
| PIGG | SLC38A7 | UBE2Z | NINL | CDHR5 | CHCHD6 |
| IQCE | GJC1 | PSMB1 | PXYLP1 | BOP1 | KIF1C |
| PRR5 | DIP2A | TTC7A | IMPDH1 | FYN | HPCAL1 |
| ZNRF3 | ARHGAP33 | DGKZ | OSBP | LRRK1 | C1orf174 |
| GPR153 | GLOD4 | EHMT1 | TBC1D10C | SND1 | PRPSAP1 |
| NUTF2 | PMVK | UNK | DIDO1 | CDK5RAP3 | OBSCN |
| TMED4 | CBFA2T3 | PEAK1 | ZNF212 | SPTAN1 | FAM47E |
| RAVER1 | PGK1 | SCAF1 | ULK3 | SOX5 | CCDC167 |
| CARM1 | SNX12 | C16orf70 | UBE2H | ARHGEF7 | URB1 |
| CUL9 | MCM7 | MECR | GOLGA6L4 | RNF40 | MXRA8 |
| FADS2 | APEX2 | GLB1L3 | OTUD7B | ERGIC1 | BOLA1 |
| TRIM41 | ELAC2 | PDCD2L | MAFG | SOGA1 | NUTM2D |
| AAMP | DHCR24 | MXD3 | TTLL12 | MATN2 | ELAVL1 |
| RASSF5 | PKM | TXNDC11 | AMBRA1 | RBFOX2 | RAB4B |
| LRWD1 | SMAD3 | FAM83H | TRPC4AP | POLDIP3 | TNS4 |
| MATR3 | ELMSAN1 | GGA3 | DENND3 | PDE6D | VILL |
| DBNL | ANXA6 | SLC16A3 | ATF6B | CPNE7 | KDM5B |
| PAPPA | SNRPN | TSC22D4 | SUN2 | ST3GAL2 | RPL35 |
| FRMPD3 | CEP164 | IGF2R | CABIN1 | LMNB2 | RGS12 |
| HSF1 | NRBP2 | RGS6 | BEGAIN | MAPKAPK2 | FMNL1 |
| PEX5 | ZXDC | LPIN1 | COPG1 | CACNA1H | IGF2BP1 |
| HARS | ARRDC1 | EXTL3 | MBTPS1 | CD247 | ELOF1 |
| GALNT2 | OAZ2 | ARHGAP11B | ADAMTS7 | GNA15 | PKIG |
| FAM184B | AP3D1 | BAHCC1 | ATXN7L3 | TET1 | SPATA32 |
| TERT | SNRPA | KRTCAP2 | ALKBH5 | HOXB3 | TRIM28 |
| GFRA1 | NUP214 | ATG2A | APPL2 | IMMP2L | SUPT6H |
| MTHFR | BRD1 | CLASRP | PACSIN2 | PEX14 | GNAO1 |
| PHF21A | DGAT1 | CECR2 | CCDC88C | TAOK2 | G6PD |
| PLAC9 | PTTG1IP | PPP1R8 | KCNH2 | PAN2 | AP1B1 |
| POLR3E | PSKH1 | HSH2D | ASB13 | SHANK2 | PIGQ |
| NPEPPS | GRM4 | EPHB2 | POLE | ZNF324 | PLA2G4C |
| VARS2 | PDLIM7 | CSPG4 | TMEM44 | CASKIN2 | APOBR |
| HOXA3 | DFFB | AKNA | SUSD6 | DNAH17 | MED12 |
| ZNF792 | MAST1 | NIPSNAP1 | TAGLN2 | CHID1 | NCOA6 |
| MON1B | TIMM21 | TVP23A | POLG | ZNF292 | MGAT5 |
| ARF1 | TRIM5 | HIF1AN | COL6A6 | TLN1 | GTF2I |
| PHF20 | LAMTOR1 | EVL | GRAMD4 | TNRC6C | PER1 |
| AHNAK | MYL6B | WAS | CD5 | AP1M1 | CCDC69 |
| TNRC6B | WWC3 | IL6R | CTNND1 | KHSRP | CLPTM1L |
| TCF3 | KDM2B | MLEC | ZAP70 | PTPRD | GSK3A |
| SZT2 | BAIAP2 | CAMTA2 | EP300 | NBEAL2 | FLII |
| SLC22A23 | GEMIN4 | E2F1 | MCM5 | TUBGCP6 | REXO4 |
| FXYD2 | MEGF11 | TFEB | PDCD6 | PDE2A | PPP1R12C |
| HELB | NOL4L | MLLT6 | DNAJC5 | EFCAB6 | KIAA0753 |
| DCPS | FDXR | DISP1 | EMG1 | HMOX2 | GTPBP1 |
| EFNB3 | B9D1 | TEP1 | MAVS | RNF39 | LYST |
| KLC2 | SPNS3 | SYK | CHST12 | TSPAN15 | MAPT |
| NATD1 | YEATS2 | MLYCD | GNG7 | RAB40C | GGA1 |
| POLD1 | APBA1 | PRCC | ABAT | ATP2B4 | MSH5 |
| TECPR1 | RNF31 | TULP3 | DRAP1 | C1orf216 | ARHGDIA |
| ABCC1 | FOXM1 | LENG8 | XPNPEP3 | ENTPD6 | SART1 |
| AHRR | RIN3 | FBXW7 | KIAA0355 | PI4KB | SPEG |
| SPTBN4 | FSD2 | NAALADL2 | RBM33 | FOXJ3 | ZNF827 |
| HIPK2 | PLXNB2 | BRD9 | CYTH3 | MCCC1 | RANBP3 |
| ANGPTL6 | NCLN | NHP2 | ARSA | PDPR | KIAA0556 |
| ATP7B | MECP2 | SCO1 | TOP3A | CBX2 | SCUBE1 |
| MAPRE3 | TBCD | ATP2A1 | JMJD6 | KAT6A | FGD5 |
| FAM172A | SNX30 | PPP5C | DDX41 | CD3E | MAP1B |
| SAMD1 | MYDGF | SH3PXD2A | AKT1 | MAU2 | IPO13 |
| USP19 | INPP5D | CCNYL1 | SLC4A8 | KLHL29 | TMEM255B |
| COG8 | RPAP1 | PRRC2A | SOCS7 | ACAD8 | RPH3AL |
| CCDC57 | CHMP4B | MAN2C1 | FAM45A | ADCY1 | TNIP1 |
| ATG4B | LLGL1 | BRI3 | MAPKAPK3 | TMEM259 | LRRC41 |
| PDZD4 | ZNHIT1 | NUP210 | MAPKBP1 | TUB | DCUN1D2 |
| POMT1 | MAFK | NEK5 | PYGB | PLXNB1 | RASSF7 |
| LEMD2 | FAM86C1 | ZBTB38 | SYNGAP1 | FBXL2 | BICD1 |
| CASC3 | RAPGEF1 | ZNF219 | PRRC2C | SRF | MOB3A |
| SASH3 | MYPOP | ADD1 | FLYWCH1 | SAP30 | ANKRD27 |
| RUNX1 | HNRNPL | GRIN3A | UCK2 | SATB1 | PCSK7 |
| STX16-NPEPL1 | TBC1D9B | PDE4A | CPSF1 | CLK3 | DIAPH1 |
| RPL11 | GNA12 | MFSD12 | AKIRIN2 | THOP1 | DHX38 |
| FOXP4 | NDUFB10 | PKP4 | SCYL1 | CTTN | CIC |
| R3HDM2 | SEC13 | IL12RB1 | CDYL | SLC25A39 | HTT |
| QRICH1 | TRO | TAX1BP3 | HIST4H4 | BTBD11 | TRIM25 |
| FBXO44 | TTC24 | ZNF646 | SFXN5 | CELSR2 | ATP13A2 |
| WASF2 | TRIM39 | BACE2 | IL17RC | TMPRSS6 | GIT1 |
| AHCYL2 | PFKFB3 | GNG5 | CLCN2 | ZNF337 | RAB11FIP3 |
| SYMPK | SLC2A6 | CACNB1 | TYRO3 | PCIF1 | TMPRSS5 |
| UBE2R2 | UNC93B1 | MPP2 | FRMD4A | POU2F1 | SFSWAP |
| FBXW4 | STK11 | ATN1 | CLCN6 | IDUA | LRP1 |
| MYO1C | MSI2 | SSH2 | LTBP4 | LONP1 | SMAD6 |
| POLR2A | CSK | UNKL | IRF2 | STXBP2 | DCTN3 |
| HEXIM1 | NDUFAF3 | P4HB | MYRF | CCM2 | SEMA4D |
| DCAKD | HHIP | SLC7A6 | ITPK1 | DESI1 | IFNAR2 |
| COMTD1 | NPIPB15 | UBXN6 | SH3D21 | B3GAT3 | LRP10 |
| MCM3AP | SLC6A6 | DRP2 | ZFC3H1 | ELOVL6 | DHPS |
| PABPN1 | CDC42BPB | ZNF598 | ACTN1 | RBM10 | DHX58 |
| MAGED1 | RALGDS | GLRA2 | MYO18A | FBL | PANK4 |
| MAZ | BCOR | MTMR14 | PFKP | CCNL2 | ST3GAL3 |
| CCDC142 | TAPBP | KANK4 | EPHX2 | NDUFA6 | KDM7A |
| TPCN2 | POLD2 | C17orf51 | MRPL48 | RAI1 | TSPAN18 |
| MBOAT1 | PSMB10 | METTL22 | EPN2 | MRGBP | TMUB1 |
| DPF2 | SETD5 | TCTE1 | TCERG1 | ANKLE1 | PHRF1 |
| BCKDK | FBXO36 | LPCAT3 | FURIN | LRRC20 | EMID1 |
| EMC1 | SSU72 | MAPK12 | ZFAND3 | SMG6 | SLIT1 |
| ACOX3 | PTPRE | SMTN | SWI5 | SFMBT2 | LIMK2 |
| WDR20 | ANAPC5 | CACNA2D2 | ARHGAP27 | CHMP1A | SNAP47 |
| ABCG1 | TAF15 | NMRAL1 | IGSF3 | TOR1B | RPL12 |
| PNPLA6 | ENKD1 | ABHD17A | MDM4 | NAA38 | STK10 |
| SNED1 | TACC3 | BCL9 | MBD1 | NPHP4 | ZBTB48 |
| CLSTN1 | COX6B1 | ZNF831 | RNPS1 | FN3KRP | CES4A |
| RNF4 | BAZ2A | MIER2 | ADSSL1 | FILIP1 | HIC2 |
| DDX19A | TEAD1 | CTSD | GRID2IP | KMT2E | BAHD1 |
| CC2D1B | MAGEF1 | FAM129A | CNTROB | TMED9 | AP5Z1 |
| PLEKHA7 | SMCR8 | TSPYL5 | PSMA1 | SPC24 | SNAP23 |
| COA1 | ARAF | MED9 | TYK2 | INO80E | PRDM16 |
| RAD23A | TSPAN11 | PCBP1 | CDH3 | EHMT2 | CDC25B |
| GAL3ST2 | ODF2 | POLR2F | LHB | GPR137 | KMT2A |
| LRSAM1 | KDM2A | ATG7 | REEP1 | ANTXR1 | APLN |
| DPP9 | FADS3 | FZR1 | KIAA0319L | POM121C | SDR39U1 |
| PGS1 | IQSEC1 | PLEKHM2 | HUWE1 | GAK | ZNF460 |
| NFATC4 | TMEM104 | RAB44 | TMEM39B | GRID1 | CYTH2 |
| PTPRN2 | ARFGAP1 | KREMEN2 | FAM160B2 | HDAC4 | PITPNM1 |
| ARHGAP31 | CDK4 | RAB11FIP4 | DYNC1LI2 | PSMF1 | LPP |
| PTPN23 | IGLON5 | SDHA | SEC31B | SEC24B | COLGALT1 |
| TBC1D25 | HEATR4 | GNL3L | RNF220 | FDFT1 | DOT1L |
| NELFB | CIR1 | NECAB3 | KLF16 | RADIL | SLC9A3R1 |
| PASK | NLRC3 | SERF2 | AKAP1 | MED29 | VPS53 |
| FARSA | HAGH | RPL36 | AP5B1 | NGEF | APOL2 |
| EML3 | AARSD1 | GON4L | CLIC6 | PUM1 | HIRA |
| DENND4B | ALG12 | KIF22 | GLG1 | CHTF18 | UBASH3A |
| NFATC2IP | SKI | ARHGAP30 | SLC38A5 | SIPA1 | NADSYN1 |
| OPA3 | FOXO4 | ZMYM3 | GLYCTK | MAP3K12 | NFKB2 |
| ANKLE2 | PAK4 | GLIS1 | PDXDC1 | RAB1A | PHACTR4 |
| ITGB2 | OPCML | POU2F2 | ZNF467 | ADCY3 | STRADA |
| MYH7B | FKBP14 | B4GALT1 | PPIB | GLB1L2 | KATNB1 |
| CLDND2 | KMO | ICA1 | PRKCSH | CHD7 | KDM3A |
| MBD6 | ATXN1 | ARHGAP23 | MAGOH | VWA7 | BCL2L1 |
| PRPF40B | BACH2 | PIGL | LRIG2 | SLC25A22 | MAN1B1 |
| GPR108 | PLOD1 | ALKBH2 | RANGAP1 | SP9 | KIAA1024 |
| KMT2B | DGUOK | BBS12 | CTDNEP1 | SIGIRR | TELO2 |
| LPIN3 | FTO | UBXN1 | COL27A1 | GPR182 | PPRC1 |
| PRMT1 | ZNF318 | SCRIB | LRFN1 | ATL1 | IKZF1 |
| FCHO1 | RPUSD1 | SCMH1 | VPS13D | POFUT2 | PPP6R1 |
| RPS6KA2 | C9orf16 | PIP5K1C | IL17RA | TRERF1 | FOXN3 |
| ESR1 | CORO2A | FAM78A | KCNAB2 | ZNF562 | LARP4B |
| PDE6G | P2RX1 | FGD3 | AAK1 | CCND1 | AP2B1 |
| CASP4 | MED15 | SAMM50 | BTRC | TFCP2 | NPHP3 |
| FAM13A | PPP2R5D | METTL16 | DAAM2 | BAP1 | MAP1S |
| CHD3 | TRIM14 | FBLN2 | SETD1B | ABCB6 | MRPS15 |
| ESPN | C20orf27 | SCAMP3 | SBK1 | CCDC85C | CPNE5 |
| LIG1 | PPARD | POMT2 | DACH1 | TAF4 | NUMA1 |
| LMTK3 | CASZ1 | KIAA1549 | TMEM120B | RECQL4 | GRHPR |
| DDX17 | SIL1 | HMGXB3 | FNDC3B | SNAPC4 | C20orf194 |
| KIF27 | CRTC2 | FAM168A | AMPD2 | TOMM7 | BMP8B |
| TNK2 | ANKRD52 | LAMC3 | KAT8 | VASP | VGLL4 |
| TBC1D8 | GPC3 | JAM3 | PCNT | SLC31A2 | ROBO3 |
| CSNK1D | ARAP1 | SNAPC3 | HNRNPUL1 | BCR | BYSL |
| SHISA5 | SYNE1 | RBCK1 | ADGRL1 | NCOA3 | ALDOA |
| CLCN7 | PRR12 | SPATC1L | ABHD2 | KCTD17 | FLNB |
| TNRC18 | ZNRF1 | FAM227A | BDH1 | PRMT5 | KIAA0895L |
| ZNF316 | CDS2 | GRK6 | DDRGK1 | GTSE1 | GIGYF1 |
| PGPEP1 | HSPG2 | DDX39B | MZT2B | CPNE2 | TAF1 |
| MAN1C1 | STRN4 | CEP112 | TMOD2 | ATRN | ACBD4 |
| UBASH3B | POLN | PKMYT1 | ADAMTS13 | ZRANB3 | TSC1 |
| CLIC2 | USF2 | PNKD | CATSPER2 | KLHDC3 | B4GALT5 |
| BRAT1 | XYLT1 | KCNG2 | TSSK4 | SLC2A13 | PRPF6 |
| GSE1 | CLPTM1 | RBM42 | PRKX | VAMP8 | CFL1 |
| HDLBP | HCFC1 | INTS1 | PABPC1L | CMC1 | RTN4RL2 |
| FAM219A | PODNL1 | SEMA3F | NBPF14 | FRMPD4 | SPIDR |
| ARHGEF17 | NOSTRIN | ACAP1 | MED28 | C6orf106 | KAT2A |
| TBC1D24 | PDIA4 | ABCC5 | DNM1 | MADCAM1 | KLF13 |
| MINK1 | HERC1 | MAP2K6 | RAP1GAP | SNAP91 | DTD1 |
| COMT | SMARCA4 | NELFA | EDF1 | XRRA1 | ANXA11 |
| ARHGAP4 | SLC25A35 | RBL2 | C19orf71 | DTX1 |  |
| FAM69B | PC | PACS1 | KCNN1 | POMGNT2 |  |
| RAB7A | CALCOCO1 | MST1 | PKHD1 | FUS |  |
| TM9SF4 | ARHGAP44 | TAF8 | SURF6 | PLCB3 |  |
| TSHR | SFI1 | LIMD1 | TTI1 | WNK1 |  |
| ZSWIM6 | MEN1 | EFCAB5 | TARBP1 | MICAL3 |  |
| ATXN7L1 | SHANK1 | TANC2 | MEGF6 | UQCRQ |  |
| SMG5 | B4GALNT4 | ARL2BP | DDR1 | PCSK4 |  |
| ZDHHC8 | PSD3 | CABLES2 | ALDH2 | NCR3 |  |
| TCF7L2 | PML | PLCB2 | INPP5A | WDTC1 |  |
| COPE | AGPAT4 | ZC3H18 | NDUFS6 | SH3BGRL3 |  |
| RTEL1-TNFRSF6B | SETD3 | NID2 | RHOC | MAML1 |  |
| SLC30A8 | PSD4 | MTR | MTMR4 | MAP3K13 |  |
| NPRL3 | RALGAPA2 | FBXW5 | COL25A1 | IGF1R |  |
| RUNDC1 | USP30 | CCDC146 | C2orf68 | TSC2 |  |
| KIZ | UBR4 | XXYLT1 | ARRB2 | TIMP2 |  |
| PRKACA | PLEKHA2 | FKBP8 | CPSF4 | JPH3 |  |
| EVPL | ABLIM1 | NACC1 | HERC2 | DOCK2 |  |
| ST6GAL1 | HIVEP2 | CDC34 | TBC1D10A | MAP2K2 |  |
| SBF2 | EHD1 | FPGS | PIK3R6 | ZNF592 |  |
| EPHB6 | ADAR | VDR | CD151 | ACLY |  |
| RBM15B | MTOR | ASS1 | NDUFV3 | MOB2 |  |
| OTOF | ANKFY1 | TOP3B | DNASE2B | AMFR |  |
| EHBP1L1 | KIFC2 | FBXO46 | AMOTL1 | GPR87 |  |
| ZNF814 | MGRN1 | CARD14 | MYO1G | FBXO41 |  |
| RTEL1 | PRDM2 | TRIT1 | TAF11 | MARK4 |  |
| ZMIZ1 | FNBP1 | NOC2L | SLC45A4 | DLG4 |  |
| GALNT13 | PDXK | CTBP1 | VARS | PTPRF |  |
| WDR59 | NFATC1 | UQCR10 | MYO9A | EHD4 |  |
| PTOV1 | SCLY | MED13L | PXDN | PTMS |  |
| MYH10 | ABCA4 | SNRNP70 | SF3A2 | PARP12 |  |
| ESPL1 | NONO | LSS | NFRKB | ABCD4 |  |
| HS6ST1 | MYO19 | LRIG1 | MTA1 | NADK |  |
| PIWIL4 | PPME1 | FOXJ2 | TANGO2 | GAPVD1 |  |
| ABR | USP11 | ANKRD11 | CDK10 | FAM129B |  |
| ACAP3 | ATG9A | NFATC2 | FPGT-TNNI3K | CMTR1 |  |
| P3H1 | MROH1 | GORASP1 | SUPT5H | GLYR1 |  |
| MKNK2 | NOVA1 | GRK4 | GALNT16 | EIF6 |  |
| B3GAT1 | AIP | COX19 | NOTCH1 | CORO1C |  |
| PTMA | AKAP8L | LDB1 | CSAD | PWWP2B |  |
| SPON2 | ABI3BP | GAA | SLC39A11 | POLH |  |
| CGN | RHOH | KDM5C | HDAC11 | PKN1 |  |
| RREB1 | GRK5 | AZIN2 | DDB2 | USP42 |  |
| SIK2 | RPS25 | MIDN | FUZ | CACNA1I |  |
| FBXO10 | CACNA1A | TEKT5 | BOD1L1 | ZCCHC14 |  |
| CLK2 | TNXB | ZC3H3 | KCNIP3 | KIF13B |  |
| EBF4 | AEBP1 | ZFP90 | NDUFS5 | MAP4 |  |
| PHYKPL | CCNK | ACTN4 | ATP11A | LMBR1L |  |
| TRMU | SCG5 | SNTA1 | PIEZO1 | OTUD5 |  |
| ULK1 | RPL7L1 | C18orf32 | ITPR3 | GPATCH2L |  |
| SVIL | PER2 | RASGEF1C | NR6A1 | ADGRB1 |  |
| DPYSL2 | ANXA4 | TMEM63A | SIN3A | FBXO34 |  |
| ZBTB7A | ARHGAP17 | CIZ1 | SPOCK2 | INPPL1 |  |
| ACSS1 | THBS4 | PKD1 | ZDHHC24 | CUX1 |  |
| PIP4K2B | FAM222A | CBX6 | ANKS1A | PLXNA3 |  |
| CTDSPL | SRGAP3 | ADAMTS17 | ASIC1 | MYBBP1A |  |
| PRR14L | HMBOX1 | TRPM2 | MCF2L | SYNRG |  |
| STK40 | TMEM63C | EML2 | BRD3 | CD3D |  |
| STK25 | CLIP2 | TNKS | DCAF8 | ZNF687 |  |
| CORO1A | CHD5 | ACTR1B | NOL6 | CYB561A3 |  |
| REPS2 | PLEKHO2 | STXBP1 | STMN3 | CUEDC1 |  |
| PNKP | NDUFA3 | FAM53A | ATXN7L3B | SNX27 |  |
| CHD8 | ELK1 | FBXO21 | HIVEP3 | WDR62 |  |
| FAM160A2 | MAST2 | NDRG1 | BRF1 | FAT1 |  |
| SYTL1 | ZSCAN25 | CAMTA1 | PBLD | RNF24 |  |
| AK4 | GRAMD1A | CCDC93 | STX5 | OLFM2 |  |
| KAZN | DHRS4 | SLC25A16 | ILF3 | SUGP1 |  |
| GRIPAP1 | CROCC | SLC26A11 | CD6 | ST3GAL1 |  |
| ASH1L | LMTK2 | DNAJC12 | ITGA1 | PCGF3 |  |
| LRP5 | MRPL23 | TOLLIP | IDH3G | PRPF8 |  |
| ELP2 | CDIP1 | RPTOR | BLMH | PPP1CA |  |
| N4BP3 | SNRPA1 | PLEKHM3 | ACACA | TNFRSF12A |  |
| EEF2K | MYH11 | GUK1 | RHPN1 | ALDH16A1 |  |
| ZNF236 | PDE1A | RHPN2 | RAB43 | GLDC |  |
| GMPPB | AGAP2 | DNPH1 | BECN1 | MUM1 |  |
| ZNF768 | HECTD3 | AKR1A1 | ZHX2 | KCNC4 |  |
| SYDE2 | EXOC4 | WDR27 | RNF213 | CES3 |  |
| JARID2 | MAMLD1 | HKR1 | POLM | HK2 |  |
| PMM2 | RNF145 | RRBP1 | TXLNA | CXXC1 |  |
| TTBK2 | SCARB1 | TARDBP | CX3CR1 | WSB1 |  |
| WNT3 | PHF1 | HMGA1 | BSG | DNAH7 |  |
| NBPF9 | NFE2L1 | LZTS1 | SERPINF1 | GYS1 |  |
| GRIN2C | NMT1 | KCTD15 | PITPNM2 | FAM207A |  |
| CCS | ARIH2 | SERHL2 | NCAPH2 | TCF25 |  |
| CIT | CLIP3 | SLA2 | ZNF384 | USP5 |  |
| VIPR1 | WBP2 | GAS2L1 | SAP130 | ABCD1 |  |
| NUP93 | ZFP36L2 | CREB3L1 | ENO1 | XYLT2 |  |
| TPCN1 | WDR19 | ZNF397 | ZBTB25 | RNF169 |  |
| ABTB2 | RNF126 | GAS7 | FBXL20 | BRSK1 |  |
| GBE1 | C12orf43 | IQGAP3 | AK2 | AHDC1 |  |
| ADAM22 | RELA | KLHDC4 | ACSF3 | DNMT3A |  |

**Supplementary file 2** The 22 hit compounds.

**Table. Hit compounds that potentially bind to IGF2BP2**

| **NO.** | **Hit compound** | **CS** | **Affinity(kcal/mol)** |
| --- | --- | --- | --- |
| 1 | ZINC08443691 | 438455-49-3 | 13.5 |
| 2 | ZINC08397484 | 547710-10-1 | 12.4 |
| 3 | ZINC08426863 | 720676-06-2 | 12.3 |
| 4 | ZINC02086445 | 547761-92-2 | 11.9 |
| 5 | ZINC02190780 | 371144-50-2 | 11.8 |
| 6 | ZINC08383903 | 55386-38-4 | 11.8 |
| 7 | ZINC08451433 | 956206-57-8 | 11.7 |
| 8 | ZINC04066571 | 304683-22-5 | 11.7 |
| 9 | ZINC02066103 | 301207-96-5 | 11.6 |
| 10 | ZINC04636142 | 496786-57 | 11.6 |
| 11 | ZINC08455667 | 548469-49-4 | 11.6 |
| 12 | ZINC08456283 | 500127-92-4 | 11.6 |
| 13 | ZINC08398688 | 330971-18-1 | 11.6 |
| 14 | ZINC02060570 | 63464-59-5 | 11.6 |
| 15 | ZINC02065222 | 499189-17-2 | 11.6 |
| 16 | ZINC00669682 | 438453-11-3 | 11.5 |
| 17 | ZINC08387227 | 663197-68-0 | 11.5 |
| 18 | ZINC08432580 | 547747-81-9 | 11.5 |
| 19 | ZINC08386110 | 639057-50-4 | 11.4 |
| 20 | ZINC08434835 | 139299-40-4 | 11.4 |
| 21 | ZINC08415218 | 421567-38-6 | 11.3 |
| 22 | ZINC08432683 | 547748-36-7 | 11.3 |
